# Latent Epstein-Barr virus infection collaborates with Myc over-expression in normal human B cells to induce Burkitt-like Lymphomas in mice

**DOI:** 10.1371/journal.ppat.1012132

**Published:** 2024-04-15

**Authors:** Jillian A. Bristol, Scott E. Nelson, Makoto Ohashi, Alejandro Casco, Mitchell Hayes, Erik A. Ranheim, Abigail S. Pawelski, Deo R. Singh, Daniel J. Hodson, Eric C. Johannsen, Shannon C. Kenney

**Affiliations:** 1 Department of Oncology, McArdle Laboratory for Cancer Research, School of Medicine and Public Health, University of Wisconsin Madison, Madison, Wisconsin, United States of America; 2 Department of Pathology, University of Wisconsin-Madison, Madison, Wisconsin, United States of America; 3 Wellcome-MRC Cambridge Stem Cell Institute, University of Cambridge, Cambridge, United Kingdom; 4 Department of Medicine, School of Medicine and Public Health, University of Wisconsin-Madison, Madison, Wisconsin, United States of America; Brigham and Women’s Hospital, UNITED STATES

## Abstract

Epstein-Barr virus (EBV) is an important cause of human lymphomas, including Burkitt lymphoma (BL). EBV+ BLs are driven by Myc translocation and have stringent forms of viral latency that do not express either of the two major EBV oncoproteins, EBNA2 (which mimics Notch signaling) and LMP1 (which activates NF-κB signaling). Suppression of Myc-induced apoptosis, often through mutation of the TP53 (p53) gene or inhibition of pro-apoptotic BCL2L11 (BIM) gene expression, is required for development of Myc-driven BLs. EBV+ BLs contain fewer cellular mutations in apoptotic pathways compared to EBV-negative BLs, suggesting that latent EBV infection inhibits Myc-induced apoptosis. Here we use an EBNA2-deleted EBV virus (ΔEBNA2 EBV) to create the first *in vivo* model for EBV+ BL-like lymphomas derived from primary human B cells. We show that cord blood B cells infected with both ΔEBNA2 EBV and a Myc-expressing vector proliferate indefinitely on a CD40L/IL21 expressing feeder layer *in vitro* and cause rapid onset EBV+ BL-like tumors in NSG mice. These LMP1/EBNA2-negative Myc-driven lymphomas have wild type p53 and very low BIM, and express numerous germinal center B cell proteins (including TCF3, BACH2, Myb, CD10, CCDN3, and GCSAM) in the absence of BCL6 expression. Myc-induced activation of Myb mediates expression of many of these BL-associated proteins. We demonstrate that Myc blocks LMP1 expression both by inhibiting expression of cellular factors (STAT3 and Src) that activate LMP1 transcription and by increasing expression of proteins (DNMT3B and UHRF1) known to enhance DNA methylation of the LMP1 promoters in human BLs. These results show that latent EBV infection collaborates with Myc over-expression to induce BL-like human B-cell lymphomas in mice. As NF-κB signaling retards the growth of EBV-negative BLs, Myc-mediated repression of LMP1 may be essential for latent EBV infection and Myc translocation to collaboratively induce human BLs.

## Author summary

EBV is a human gamma herpesvirus that causes infectious mononucleosis and cooperates with translocation of the Myc oncogene to induce Burkitt lymphoma (BL). EBV-infected BLs are derived from germinal center B cells and contain highly latent forms of EBV infection that do not express the two major EBV oncogenes, EBNA2 and LMP1. It has been difficult to model how latent EBV infection cooperates with Myc translocation to form BLs, as EBV cannot transform B cells *in vitro* in the absence of EBNA2 or LMP1. Here we show that human cord blood B cells co-infected with both an EBNA2-deleted EBV mutant and a Myc-expressing retrovirus form BL-like tumors in immunosuppressed mice, while cells infected with EBNA2-deleted EBV alone or the Myc-expressing retrovirus alone do not. These BL-like tumors express many cellular genes over-expressed in human BLs (including genes associated with a germinal center B cell phenotype) and do not express either EBNA2 or LMP1. Furthermore, we show that Myc blocks expression of the highly immunogenic LMP1 protein in BLs by inhibiting the transcriptional activator, STAT3. This new model should be useful for dissecting the mechanisms by which latent EBV infection and Myc translocation cooperatively induce human BLs.

## Introduction

Epstein-Barr virus (EBV) is a gamma herpesvirus that causes a variety of different types of lymphomas in humans, including diffuse large B cell lymphomas, Burkitt lymphoma (BL), Hodgkin lymphoma and post-transplant lymphoproliferative disease [1]. EBV transforms human B cells *in vitro* into immortal lymphoblastoid cell lines (LCLs) that have “type III” viral latency and express each of the 9 different EBV latency proteins. The two major EBV oncoproteins, EBNA2 and LMP1, mimic constitutively-active Notch and CD40 signaling, respectively, and are essential for EBV transformation of B cells *in vitro*. EBNA2 drives transcription of the latent viral genes (including LMP1) during type III latency and induces cellular proliferation by activating cellular genes such as cyclin D2 and Myc, while LMP1 induces canonical and non-canonical NF-κB signaling [2,3]. However, EBV-infected lymphomas in humans often have more stringent (and less immunogenic) forms of EBV latency, in which EBNA2 expression is turned off and fewer viral proteins are expressed. As stringent forms of EBV latency cannot transform B cells *in vitro*, it has been difficult to model how EBV promotes human tumors such as Burkitt lymphoma in which neither EBNA2 nor LMP1 are expressed.

Both EBV+ and EBV-negative BLs have Myc translocations, and excessive Myc expression is the main driver of BL [4]. EBV+ BLs usually have type I viral latency, in which only a single viral protein, EBNA1, is expressed, along with the virally encoded microRNAs and the viral small nuclear RNAs (EBERs) [5]. Approximately 15% of human BLs have “Wp-restricted” latency, in which a larger number of latency proteins are expressed, including EBNA1, a truncated form of EBNA-LP, EBNA3A/3B/3C, and BHRF1. Human BLs with Wp-restricted latency are infected with naturally occurring EBV strains containing deletions that remove the EBNA2 gene, and a portion of the EBNA-LP protein. Such tumors express the EBNA-LP, EBNA3A/3B/3C and BHRF1 genes under the control of the Wp EBV latency promoter [6,7]. EBV+ BLs are particularly common in children living within the “malaria belt” region of Africa, and there is evidence that malaria infection cooperates with EBV infection to promote these tumors [8,9]. In contrast, only 25–40% of BLs in western countries are EBV-positive [10].

In comparison to uninfected BLs, EBV-infected BLs contain fewer cellular mutations in genes involved in apoptosis [11], suggesting that latent EBV infection provides an essential survival advantage to EBV+ BLs by inhibiting Myc-induced apoptosis. However, exactly which EBV-encoded latency genes/RNAs cooperate with Myc over-expression to induce EBV+ human BLs is not completely understood. Myc activates expression of the pro-apoptotic BCL2L11 (BIM) gene in B cells [12,13], and Myc over-expression in the transgenic Eμ-Myc mouse model cannot induce B cell lymphomas without additional cellular mutations (in genes such as TP53) that prevent apoptosis and/or senescence in response to Myc [14]. Several different EBV genes/RNAs expressed in human BLs with Wp-restricted and/or type I latency may inhibit Myc-induced apoptosis by complementary mechanisms. For example, the EBV EBNA3A and EBNA3C proteins collaboratively inhibit BIM expression [15], and the BHRF1 protein, a BCL2 homologue, more globally prevents apoptosis [16]. Furthermore, virally-encoded microRNAs (“BARTs”) inhibit apoptosis by reducing expression of cellular genes such as caspase 3 [14,17,18] and the EBNA1 protein decreases p53 stability by inhibiting its interaction with USP7 [19]. In addition, expression of the EBV LMP2A protein collaborates with transgenic Myc expression to induce B cell lymphomas in mice [20], although it is not clear that human EBV+ BLs have significant LMP2A protein expression. Of note, the Kempkes lab previously showed that a normal human B cell line infected with an EBNA2-deleted (P3HR1) EBV strain can indefinitely proliferate and be switched to a Myc-driven “BL-like” phenotype when EBNA2 expression is turned off and high level Myc expression is induced [21,22]. As this cell line (P493-6) has Wp-restricted latency, latent EBV infection appears sufficient to collaborate with Myc over-expression *in vitro* to transform human B cells in the absence of EBNA2, LMP1 or LMP2A expression. However, whether this is also the case for Myc-driven lymphomas *in vivo* has not been examined.

In contrast to most types of human B cell lymphomas (including EBV-infected lymphomas that express the EBV LMP1 oncoprotein [23]), reduced NF-κB and STAT3 activity is a hallmark of human Burkitt lymphomas [24–26]. NF-κB activity functions as a tumor suppressor in BL tumors, and high level Myc expression in B cells inhibits both the canonical and non-canonical forms of NF-κB signaling [27,28]. Although NF-κB signaling is often associated with reduced apoptosis, in the context of BLs activation of NF-κB signaling enhances Myc-induced apoptosis by increasing expression of the death receptor, Fas [28]. Loss of the NF-κB2 (p100/52) component of NF-κB accelerates lymphoma development in Eμ-Myc transgenic mice [29], while activation of the IKK2/NF-κB pathway inhibits lymphoma formation [28]. Likewise, expression of LMP1 inhibits the growth and tumorigenicity of EBV-negative human BL cells *in vitro* [30]. Thus, high level LMP1 expression may be incompatible with the formation of Myc-driven GC-derived BLs, although low level Myc expression collaborates with LMP1 and EBNA2 to form EBV-transformed LCLs *in vitro* with a plasmablast-like phenotype.

However, it is not yet fully understood how LMP1 expression is turned off in EBV-infected BLs. The EBV LMP1 promoters and Cp promoter (which drives EBNA2 expression) are extensively methylated in human BLs, and methylation of the proximal ED-L1 LMP1 promoter inhibits EBNA2-mediated activation of LMP1 [31,32]. DNA methylation of the Cp and LMP1 promoters in BL cells is initiated by the *de novo* DNMT3B DNA methyltransferase and maintained by the DNMT1/UHFR1 maintenance DNA methylation complex [33]. LMP1 expression can be reactivated (in the absence of concomitant EBNA2 expression) in EBV+ BL cell lines by treatment with IL21, an effect potentially mediated by STAT3 activation [34–36]. It has not been possible to study how LMP1 expression can be turned off completely in EBV-infected normal human B cells, as LMP1 is required for the establishment of long-term lymphoblastoid cell lines. The relatively high level of Myc expression (induced by EBNA2) that occurs early after EBV infection in normal B cells has been shown to reduce (but not eliminate) LMP1 levels, although the mechanism for this effect is unclear [37]. The P493-6 B cell line, which is infected with an EBNA2-deleted (P3HR1) EBV strain, expresses LMP1 and maintains type III latency when complemented with an estrogen-driven EBNA2 protein, but loses LMP1 expression when EBNA2 is turned off and high level Myc expression is induced [21,22]. Nevertheless, it is unclear whether loss of LMP1 expression in the Myc-expressing P493-6 cells is simply due to the loss of EBNA2 activity, or whether high level Myc expression also contributes.

Although patient-derived EBV+ BLs can be grown in culture and studied in xenograft models, there is currently no model for creating EBV-infected BL-like lymphomas from primary human B cells. Here we show that an EBNA2-deleted EBV mutant (ΔEBNA2 EBV) cooperates with Myc over-expression to form BL-like lymphomas that lack LMP1 expression in NSG mice. Furthermore, we demonstrate that Myc blocks LMP1 expression both by decreasing STAT3 expression and Src-mediated STAT3 activation, and by increasing expression of the DNMT3B and UHRF1 DNA methylation machinery. This is the first demonstration that latent EBV infection combined with Myc over-expression in normal human B cells is sufficient to induce Burkitt-like lymphomas in mice.

## Results

### Human cord blood B cells infected with an EBNA2-defective EBV mutant (ΔEBNA2 EBV) grow indefinitely on a CD40L/IL21 expressing feeder cell line

**Fig 1:**
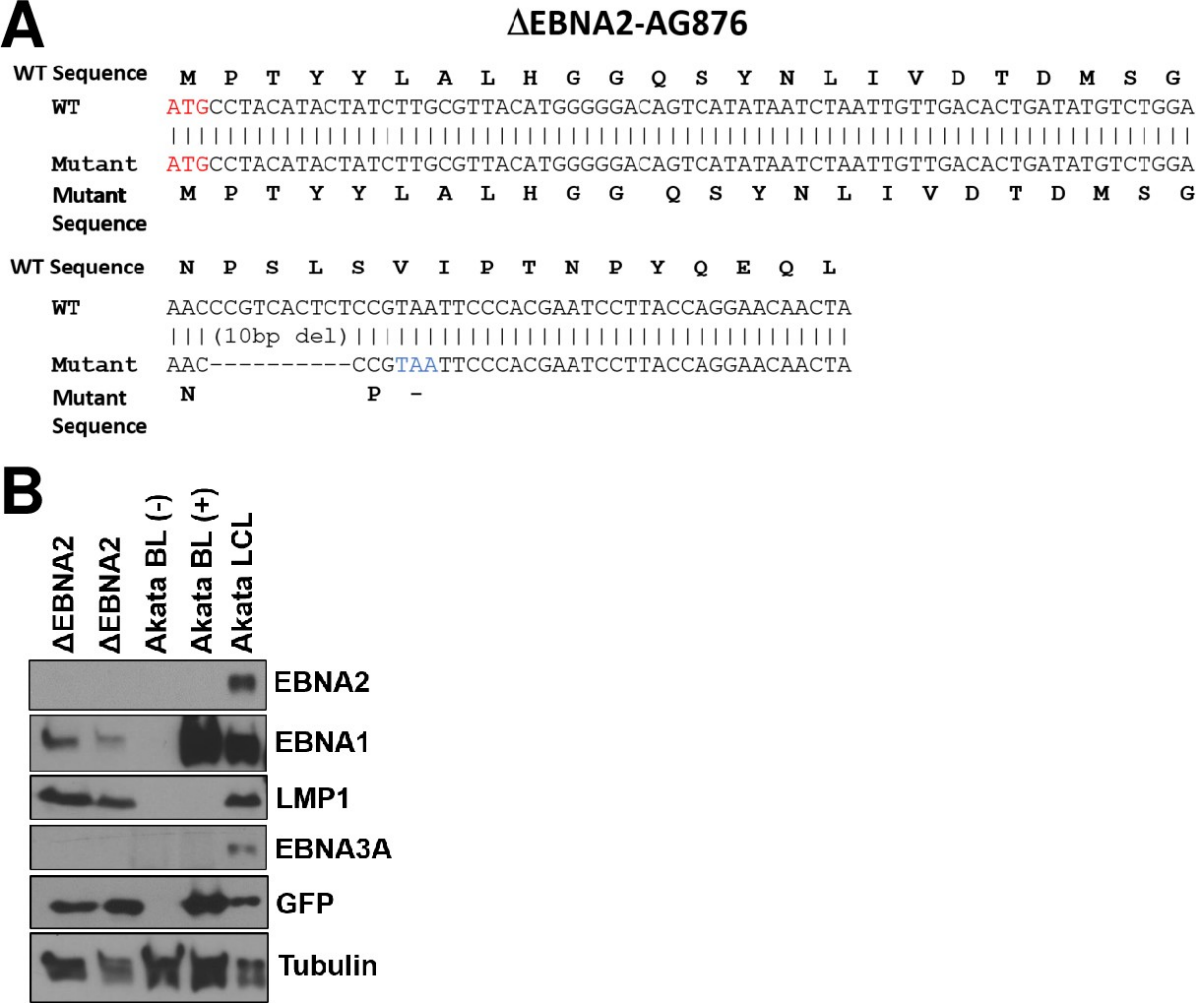
Construction of an EBNA2-deleted EBV mutant (ΔEBNA2 EBV). **A.** CRISPR/Cas9 gene editing was used to insert a 10 bp deletion in the EBNA2 gene of the AG876 EBV strain. The nucleotide sequences deleted, and the resultant translated EBNA2 protein, are shown. **B.** Protein extracts of human cord blood B cells stably infected with the ΔEBNA2 EBV mutant and maintained on a CD40L/IL21 expressing feeder layer were examined by immunoblot to assess expression of the EBV latency proteins EBNA2, EBNA1, LMP1, and EBNA3A and the GFP protein as indicated. EBV positive and EBV negative Akata Burkitt lymphoma cell lines and an LCL infected with Akata EBV (containing the GFP gene inserted into the viral BXLF1 gene locus) serve as negative and positive controls for expression of various EBV proteins and GFP.

To construct an EBV mutant virus that cannot express EBNA2 but does not remove sequences of other EBV proteins such as EBNA-LP, we used CRISPR/Cas9 technology to create an out-of-frame mutation in the EBNA2 gene (starting at amino acid 25) in the AG876 EBV strain (**Fig 1A**) as described in the Methods. EBV-infected HeLa cell clones containing only the mutant (and not wild-type) EBNA2 gene were transfected with the EBV immediate-early BZLF1 and BRLF1 genes to induce lytic viral reactivation, and the infectious virions produced were used to infect human cord blood cells cultured on an irradiated feeder cell layer (YK6-CD40lg-IL21) expressing CD40 ligand and soluble IL21 as previously described [38]. Uninfected or ΔEBNA2 EBV-infected cells were passaged and expanded as needed on the feeder layer. Similar to previous studies using primary human tonsil germinal center (GC) B cells on the same feeder layer [38], the uninfected cord blood B cells initially proliferated on the feeder layer but subsequently died within 1 month. In contrast, cord blood B cells derived from two different donors (“Donor 1” and “Donor 2”) resulted in long term EBV-infected B cell lines that can be maintained indefinitely on the feeder layer (but not off the feeder layer) when infected with the ΔEBNA2 EBV mutant, although the production of such lines is much less efficient compared to cells infected with the wild-type EBV. Both Donor 1 and Donor 2 ΔEBNA2 EBV-infected cord blood lines express Kappa, but not Lambda, light chain, suggesting that they are clonal or oligoclonal.

To confirm that ΔEBNA2 EBV-infected cord blood (CB) B cell lines lack EBNA2 expression, immunoblot analysis was performed in CB cells infected with ΔEBNA2 EBV. As shown in **Fig 1B,** ΔEBNA2 EBV-infected cells (grown on the feeder layer) express EBNA1 and LMP1, but not EBNA2 or EBNA3A, indicating the cells have “type II”-like viral latency (EBNA1+/EBNA2-neg, LMP1-pos). The expression of LMP1 in ΔEBNA2 EBV-infected CB B cells grown on the CD40L/IL21 expressing feeder layer is consistent with the known ability of IL21 to induce LMP1 expression in the absence of EBNA2 [34,35]. Together, these *in vitro* results indicate that infection with ΔEBNA2 EBV is sufficient to confer long-term proliferation potential to primary cord blood B cells on the IL21/CD40L expressing feeder layer even in the absence of any cooperating oncogenes.

### ΔEBNA2 EBV cooperates with Myc over-expression to induce lymphomas in NSG mice

**Fig 2:**
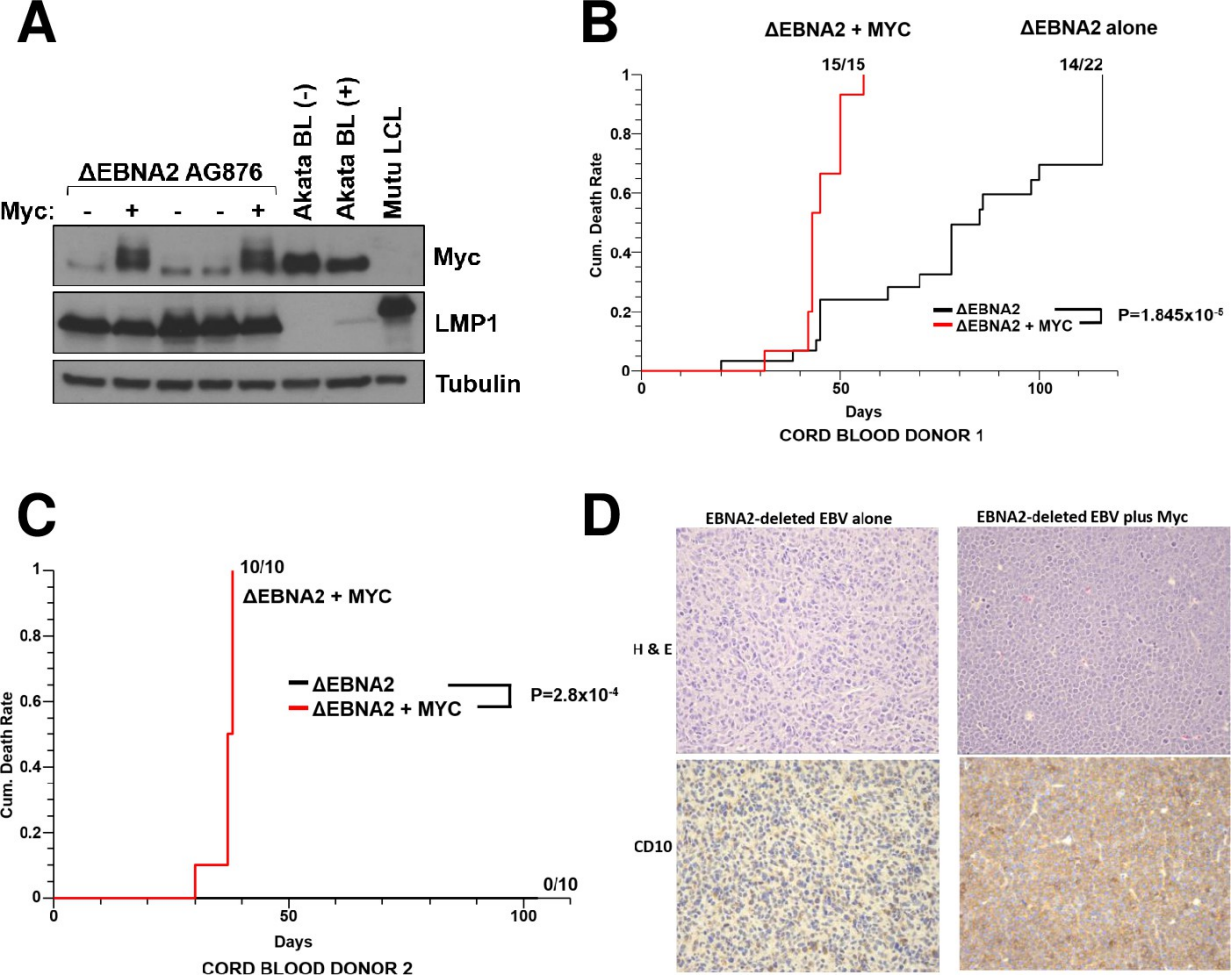
Cord blood B cells co-infected with ΔEBNA2 EBV and a Myc vector cause more lymphomas in NSG mice compared to B cells infected with ΔEBNA2 EBV alone. **A.** Human cord blood B cells stably infected with the ΔEBNA2 EBV mutant were co-infected with or without a retrovirus expressing mouse wild-type Myc protein as indicated and maintained on a CD40L/IL21 expressing feeder layer without antibiotic selection. One month after retrovirus infection, protein extracts were harvested and immunoblot analysis was performed to assess expression levels of Myc, LMP1, and tubulin as indicated. EBV-negative and EBV-positive BL cells (Akata) serve as a positive control for elevated Myc expression. **B and C**. NSG mice were injected subcutaneously with 10 million cord blood B cells (suspended in Matrigel) infected with ΔEBNA2 EBV alone, or co-infected with ΔEBNA2 EBV and a Myc expressing vector. Donor 1 cells (used in **B**) were co-infected with a wild-type mouse Myc expressing retrovirus, while Donor 2 cells (used in **C**) were co-infected with a retrovirus expressing a mutant human Myc (T58A) protein. The number of animals sacrificed with tumors at various time points after injection of cells is shown. The p-values were calculated using the Kruskal-Wallis method and program M-Stat 7.0. **D.** Tumors were paraffin-fixed and H&E staining performed to examine tumor morphology and IHC staining performed to examine expression of the germinal center B cell marker, CD10.

To determine if Myc over-expression in human CB B cells cooperates with ΔEBNA2 EBV infection to form B-cell lymphomas in NSG mice *in vivo*, and/or alters the phenotype of ΔEBNA2 EBV-infected lymphomas, ΔEBNA2 EBV-infected CB B cells were co-infected with a Myc-expressing retrovirus as described in the Methods. ΔEBNA2 EBV-infected “Donor 1” CB cells were co-infected with a retrovirus vector expressing the wild-type mouse Myc gene, and ΔEBNA2 EBV-infected “Donor 2” CB cells were co-infected with a retrovirus vector expressing a human Myc gene containing a T58A mutation previously shown to inhibit Myc-induced BIM expression [12]. Similar results were obtained using both Myc vectors. CB cells co-infected with ΔEBNA2 EBV and the Myc vectors outgrew the cells infected with ΔEBNA2 EBV alone on the feeder layer system even in the absence of any drug selection. Similar to previous results using primary human tonsil GC B cells [38], CB B cells infected with the Myc-expressing vectors alone did not result in long term lines, presumably due to Myc-induced apoptosis. To examine levels of Myc expression, immunoblot analysis was performed on CB cell extracts harvested from cells infected with ΔEBNA2 EBV alone or co-infected with ΔEBNA2 EBV plus a Myc retrovirus vector. As shown in **Fig 2A,** CB cells co-infected with ΔEBNA2 EBV and a Myc vector express Myc at similar levels found in human BL cell lines, and at a level much higher than that which occurs in wild-type EBV-transformed lymphoblastoid cell lines (LCLs) with type III latency. Expression of the EBV latency protein LMP1 was not altered by Myc co-expression when CB cells were grown *in vitro* on the feeder layer.

We next injected NSG mice subcutaneously in the flank with 10 million CB cells (suspended in Matrigel) infected with ΔEBNA2 EBV alone, or the combination of ΔEBNA2 EBV and a Myc expressing vector. Similar results were obtained using either of the two Myc vectors. As shown **in Fig 2B**, 15/15 mice injected with CB “Donor 1” cells containing both ΔEBNA2 EBV and wild-type mouse Myc protein developed tumors by day 50, whereas only 14/22 mice injected with “Donor 1” CB cells infected with ΔEBNA2 EBV alone developed tumors by day 120, and the latter tumors occurred at later time points. Similarly, 10/10 animals injected with CB “Donor 2” cells containing both ΔEBNA2 EBV and the human Myc T58A mutant protein developed tumors by day 50, whereas 0/10 mice injected with “Donor 2” CB cells infected with ΔEBNA2 EBV alone developed lymphomas by day 120 (**Fig 2C**). Tumors obtained from Donor 1 were also paraffin fixed and examined by microscopy. Lymphomas infected with ΔEBNA2 EBV alone resemble diffuse large B cell lymphomas with large polymorphous B cells, including some multinucleated cells, while tumors infected with both ΔEBNA2 EBV and Myc are composed of homogenous, medium-sized B cells with distinct cytoplasmic borders and numerous mitotic figures, closely mimicking human BL (**Fig 2D**). Thus, over-expression of Myc clearly cooperates with ΔEBNA2 EBV to allow human cord blood B cells to form lymphomas in NSG mice, although there is some donor variability in the ability of cord blood cells infected with ΔEBNA2 EBV alone to form tumors.

### ΔEBNA2 EBV plus Myc lymphomas resemble human BLs, turn off LMP1 expression and have low BIM expression and wild-type p53

**Fig 3:**
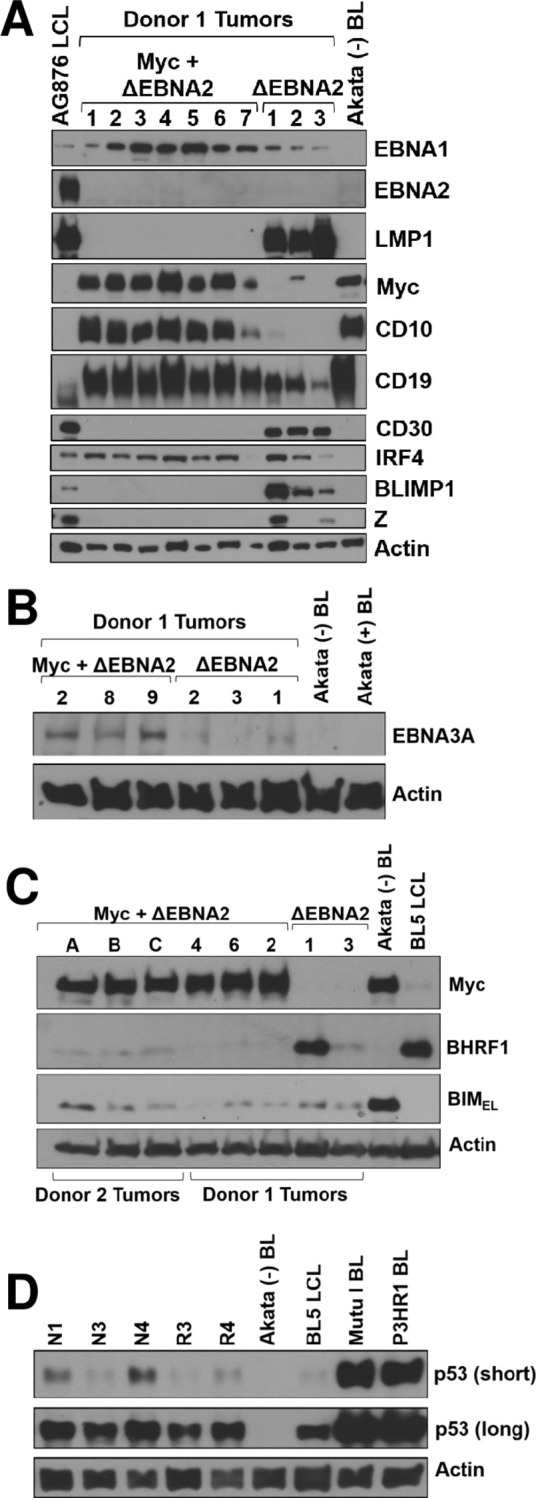
Cord blood B cells co-infected with ΔEBNA2 EBV and a Myc retrovirus cause Burkitt-like EBV-positive tumors that lose LMP1 expression. **A.** Protein extracts were harvested from tumors derived with Donor 1 cells infected with ΔEBNA2 EBV alone, or co-infected with ΔEBNA2 EBV and a Myc-expressing vector, and immunoblot analysis was performed to examine expression of various different EBV latency proteins, GFP and various cellular proteins as indicated. EBV-negative Akata BL cells serve as a positive control for Myc expression (and other cellular proteins such as CD10 expressed in BL tumors) and a negative control for EBV protein expression, while an AG876 wild-type EBV-infected LCL serves as a positive control for viral proteins expressed in type III latency. **B**. Lymphomas infected with ΔEBNA2 EBV and Myc or ΔEBNA2 EBV alone were examined for expression of the EBV EBNA3A latency protein by immunoblot. EBV-neg and EBV+ Akata BL cells serve as a negative control. **C.** Lymphomas infected with ΔEBNA2 EBV and Myc or ΔEBNA2 EBV alone were examined for expression of Myc, the EBV BHRF1 protein or the cellular BIM protein (the predominant isoform BIM_EL_ or extra-large) by immunoblot. EBV-neg Akata BL cells serve as a negative control for BHRF1 expression and a BL5 strain EBV infected LCL serves as a positive control for BHRF1 expression. Tumors derived from Donor 1 versus Donor 2 are indicated. **D.** p53 immunoblot was performed to compare p53 expression in extracts isolated from stable cell lines derived from Donor 2 ΔEBNA2 + Myc tumors (N1, N3, N4, R3, R4), the Akata (p53-deleted) BL line, the Mutu I (p53 mutant) BL line, the P3HR1 (p53 mutant) BL line, or an EBV-transformed LCL (BL5) line as indicated.

As the Donor 1 CB cells produced lymphomas in cells infected with both ΔEBNA2 EBV alone (“ΔEBNA2”), or the combination of ΔEBNA2 EBV and Myc retrovirus (“ΔEBNA2 + Myc”), we compared the phenotypes of the tumors derived from this CB donor in the presence and absence of Myc over-expression. Immunoblot analyses of tumor proteins revealed that lymphomas infected with ΔEBNA2 + Myc have high level Myc expression (similar to that in the Akata human BL cell line) and turn off expression of the EBV latent LMP1 protein in most tumors (**Fig 3A**), as well as expression of the EBV latent LMP2A protein (**S1A Fig**). Thus, as occurs in human BL tumors, the ΔEBNA2 + Myc lymphomas have a more stringent form of EBV latency (EBNA1-pos, LMP1-neg, EBNA2-neg) in comparison to the lymphomas infected with ΔEBNA2 alone, which have high level LMP1 expression. The ΔEBNA2 + Myc lymphomas also express the GC marker CD10 (MME), while ΔEBNA2 lymphomas have higher expression of CD30 (a B cell activation marker), BLIMP1 (PRDM1, a marker for plasma cell differentiation) and BZLF1 (a lytic EBV protein) in comparison to ΔEBNA2 + Myc lymphomas (**Figs 2D and 3A**).

In contrast to the type II-like latency observed when the ΔEBNA2 + Myc cord blood cell lines were grown *in vitro* on the CD40L/IL21 expressing feeder layer (**Fig 1B**), we found that the ΔEBNA2 + Myc lymphomas, in addition to losing LMP1 expression, also express high levels of the EBNA3A (**Fig 3B**) and EBNA-LP (**S1B Fig**) proteins, similar to Wp-restricted human BLs, although unlike Wp-restricted human BLs they do not express the EBV BCL2 homologue, BHRF1 (**Fig 3C**). Although commercially available antibodies do not recognize the form of EBNA3C protein expressed in type 2 EBV strains such as AG876, since EBNA3A and EBNA3C are essentially always expressed together in EBV infected cells, it is highly likely that the tumors also express EBNA3C. Because EBNA3A and EBNA3C collaboratively inhibit expression of the Myc-induced pro-apoptotic protein, BIM, *in vitro* [15], we compared BIM expression in ΔEBNA2 + Myc versus ΔEBNA2 alone lymphomas, as well as in an EBV-negative BL cell line (Akata), which has spontaneously lost the EBV genome. In contrast to EBV+ Akata cells, EBV-negative Akata cells are unable to form tumors in NSG mice and are more susceptible to apoptosis-inducing stimuli [39]. The level of BIM in the ΔEBNA2 + Myc Donor 1-derived lymphomas (which have wild-type mouse Myc) is much lower than that expressed in the EBV-negative Akata BL line, although similarly high levels of Myc are expressed (**Fig 3C**). RNA-seq analysis of Donor 1 tumors infected with ΔEBNA2 + Myc versus ΔEBNA2 alone also showed that BCL2L11 gene expression is not increased in the Myc-expressing tumors (**S1 Table**). These results suggest that one or more EBV genes/RNAs expressed in ΔEBNA2 + Myc lymphomas prevents Myc-induced BIM expression.

As p53 is commonly mutated in human BLs (particularly EBV negative BLs), we also examined the level of p53 expression in ΔEBNA2 + Myc lymphomas and determined the p53 (TP53) sequence (from our RNA-seq data) in three different ΔEBNA2 + Myc Donor 1 derived tumors as described in the Methods. The level of p53 protein expression in stable cell lines derived from the ΔEBNA2 + Myc lymphomas is similar to that expressed in EBV-transformed LCLs (**Fig 3D**), while the p53-deleted Akata BL line as expected lacks p53 expression, and the Mutu I and P3HR1 BL cell lines (both of which have mutant p53) have massively increased p53 expression. Analysis of the p53 transcript sequence obtained by RNA-seq confirmed that all three Myc-expressing tumors examined have wild-type p53 (**S2 Table**). Together, these results suggest that the relatively few EBV genes/RNAs expressed in the Myc-induced tumors are sufficient to inhibit Myc-induced BIM expression, and to substitute for the p53 mutations that often occur in human BLs.

### ΔEBNA2 + Myc lymphomas have enhanced expression of BL-associated cellular genes

**Fig 4:**
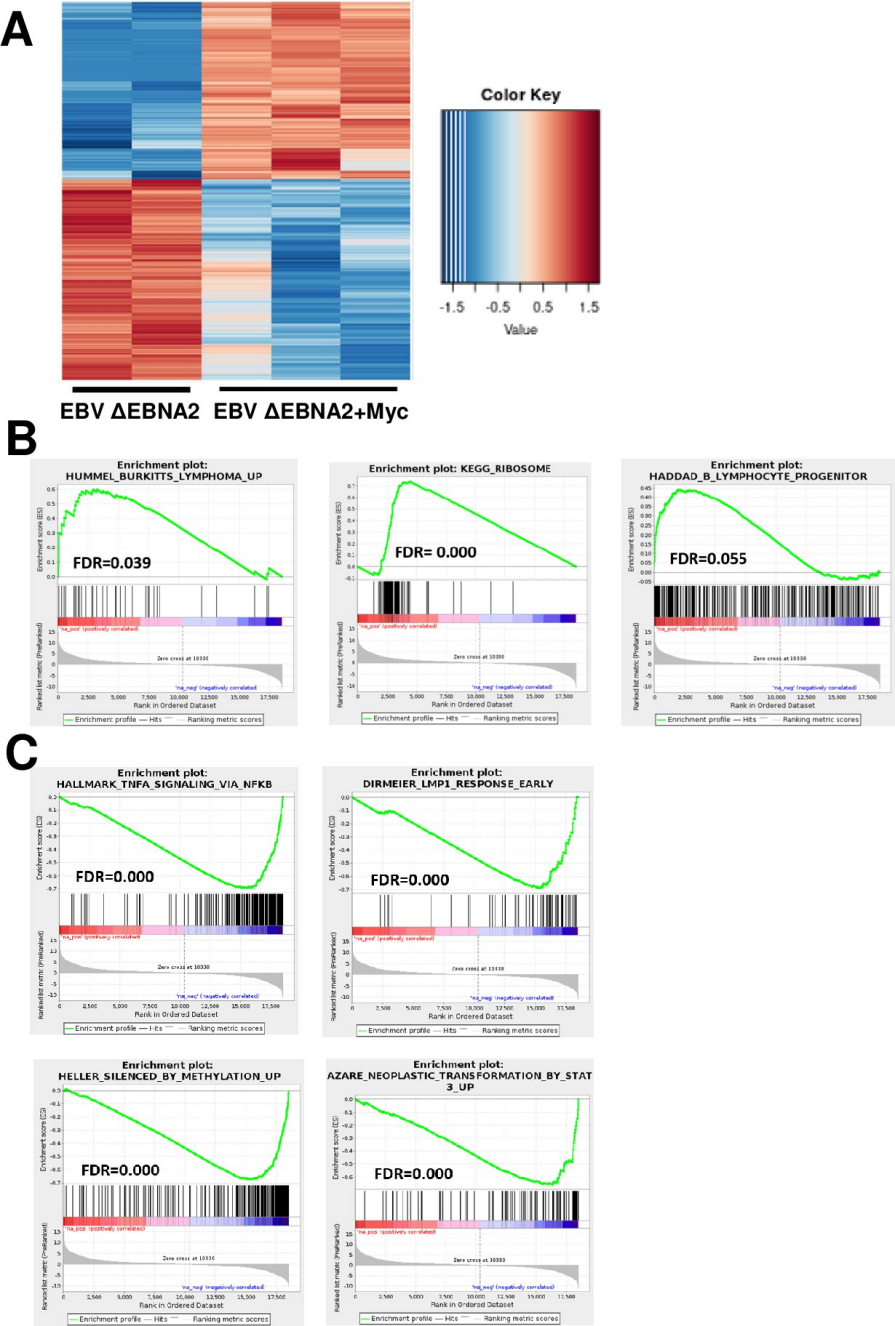
RNA-seq analysis of tumors infected with ΔEBNA2 EBV alone versus both ΔEBNA2 EBV and Myc vector reveals Burkitt lymphoma-like gene expression in the Myc positive tumors. RNA was isolated from Donor 1 derived tumors infected with ΔEBNA2 EBV alone or both ΔEBNA2 EBV and Myc vector and RNA-seq analysis was performed. **A.** A heatmap comparing cellular gene expression in tumors with and without Myc vector co-infection is shown. **B.** GSEA analysis was performed to compare gene expression in tumors with and without Myc. Selected gene sets that have up-regulated expression in the Myc-expressing tumors are shown. **C.** Selected gene sets that have down-regulated expression in the Myc-expressing tumors are shown.

To further compare cellular gene expression in lymphomas infected with ΔEBNA2 alone versus ΔEBNA2 + Myc, RNA was isolated from tumors and bulk RNA-seq analysis was performed (using tumors derived from Donor 1). As shown in **Figs 4A and S2 and S1 Table**, tumors infected with ΔEBNA2 + Myc have highly altered cellular gene expression in comparison to tumors infected with ΔEBNA2 alone. Analysis of the EBV gene expression pattern revealed that the ΔEBNA2 + Myc tumors have a lower level of lytic EBV gene expression compared to the tumors infected with ΔEBNA2 alone (**S2C Fig**), consistent with a previous study showing that Myc decreases lytic EBV gene expression in Burkitt lymphomas [40]. MIXCR analysis of the RNA-seq results (performed as described in the Methods) revealed that each of the Donor 1-derived tumors (whether infected with ΔEBNA2 only or both ΔEBNA2 + Myc) is a monoclonal B cell tumor with low IGHM expression and a rearranged Kappa light chain with a 2D-30/J3 clonotype. Thus, the ΔEBNA2-infected cord blood Donor 1 cell line used to make the tumors (with or without Myc) is likely derived from a single cell. The relatively low level of IGHM expression in Donor 1 tumors was confirmed at the protein level, although Donor 2 derived tumors express high levels of IGHM (**S2D Fig**).

GSEA analysis of RNA-seq results confirmed that Myc-expressing tumors have upregulated expression of genes in the “Hummel_Burkitt_Lymphomas_Up” gene set (**Fig 4B**), which distinguishes genes expressed in human Burkitt lymphomas versus human GC type DLBCLs [24]. Another top gene signature activated in Myc-expressing lymphomas is the “Kegg_Ribosome” signature (**Fig 4B**), consistent with Myc’s well known ability to increase expression of many ribosome components [41]. Somewhat unexpectedly, Myc-expressing tumors also have higher expression of genes in the “Haddad_B_Lymphocyte_Progenitor” signature (**Fig 4B**) [42], including a number of genes highly expressed in immature B cells. Gene sets down-regulated in the Myc-expressing lymphomas include “Hallmark_TNFA_signaling_via_NF-κB” [43] and “Dirmeier_LMP1_Response_Early” [44] (**Fig 4C**), reflecting Myc’s ability to turn down NF-κB signaling [45] as well as LMP1 expression. The “Heller_Silenced_By_Methylation_Up” gene set (which includes cellular genes in multiple myeloma that are silenced by DNA methylation and turned on by demethylating agents) [46] is also down-regulated in Myc-expressing lymphomas (**Fig 4C**), suggesting that Myc may inhibit expression of some viral and/or cellular genes by inducing DNA methylation. In addition, the “Azare_Neoplastic_Transformation_By_STAT3_Up” [47] gene set was down-regulated in Myc-expressing lymphomas, consistent with the previously described down-regulation of STAT3 expression in human BLs [24].

### ΔEBNA2 + Myc lymphomas have enhanced expression of BL core survival proteins and GC B cell proteins

**Fig 5:**
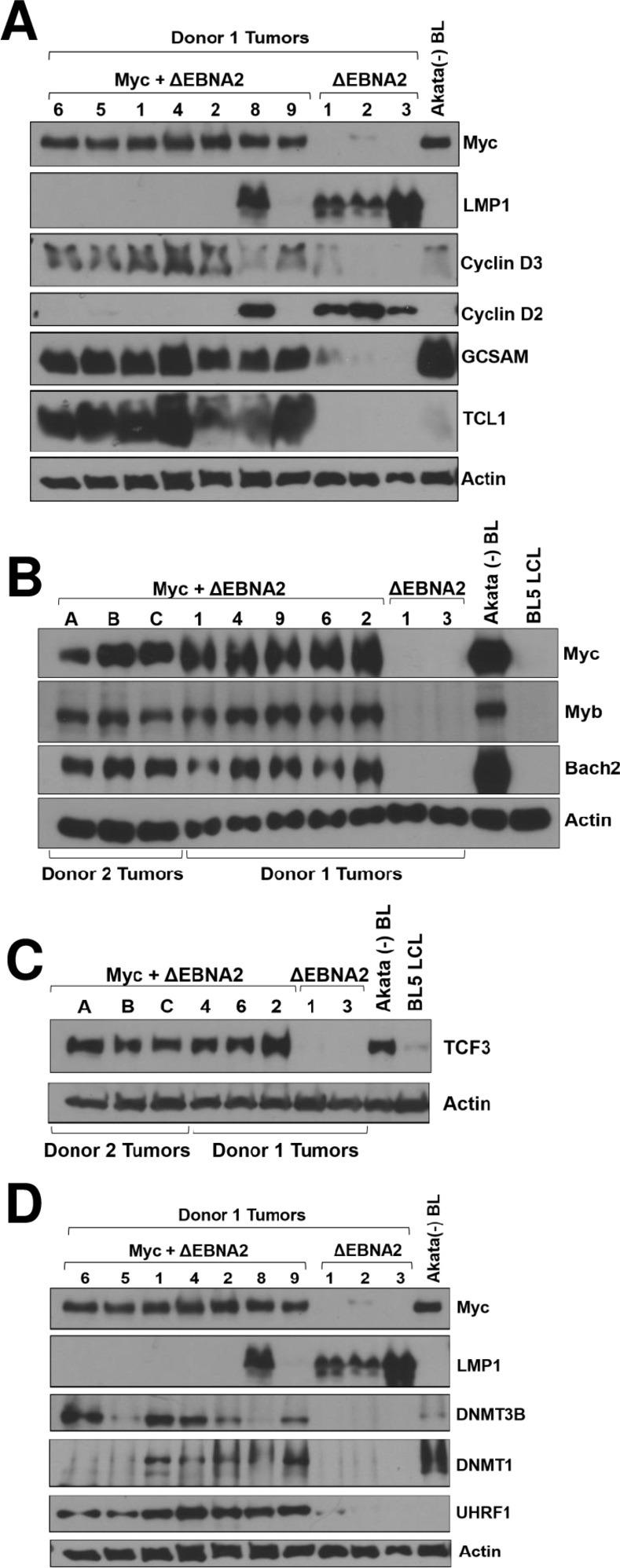
ΔEBNA2 EBV plus Myc lymphomas have enhanced expression of GC B cell proteins. Protein extracts were harvested from tumors infected with ΔEBNA2 EBV alone, or co-infected with ΔEBNA2 EBV and Myc vector, and immunoblot analysis was performed to examine expression of various cellular proteins known to be highly expressed in GC B cells and/or BL tumors as indicated. **A.** Extracts from Donor 1 derived tumors with or without Myc expression were examined for expression of Myc, the EBV LMP1 protein, cyclin D3, cyclin D2, GCSAM or TCL1 as indicated. EBV negative Akata BL cells serve as a positive control for Myc and GC B cell expressed proteins. The same actin immunoblot will be seen in Figs 5D, 6A and 7A, while the LMP1 and Myc blots will be repeated in Figs 5D and 6A. **B.** Extracts harvested from Myc expressing tumors derived from Donor 1 or Donor 2 as indicated or Donor 1 derived tumors without Myc were examined by immunoblot for expression of Myc, Myb and BACH2 as indicated. Control extracts include the EBV-negative Akata human BL cell line and a BL5 strain EBV infected LCL. **C.** Extracts harvested from Myc expressing tumors derived from Donor 1 or Donor 2 as indicated or Donor 1 derived tumors without Myc were examined by immunoblot for expression of TCF3 as indicated. Control extracts include the EBV-negative Akata human BL cell line and a BL5 strain EBV infected LCL. **D.** Extracts from Donor 1 derived tumors with or without Myc expression were examined for expression of Myc, the EBV LMP1 protein, DNMT3B protein, DNMT1 protein and UHRF1 protein as indicated. The same extracts used in Fig 5A were used for this blot and the Myc, LMP1, and actin blots from Fig 5A are reproduced here to show levels of expression in each condition.

CRISPR/Cas9 screens and other studies have identified cellular genes required for proliferation and survival of human BL cells *in vitro*, including some like CCND3 (cyclin D3) that are also required for proliferation of normal human GC B cells [48], but are not required for growth and survival of EBV-transformed lymphoblastoid cell lines [49]). Immunoblot analysis of tumor extracts confirmed that cellular proteins that are highly expressed in GC B cells, including cyclin D3, GCSAM, TCL1, Myb, TCF3 and BACH2 are expressed at higher levels in the ΔEBNA2 + Myc lymphomas in comparison to the ΔEBNA2 alone lymphomas (**Fig 5A–5C**). The transcription factor TCF3, which is required for tonic B cell receptor signaling, is an essential survival factor for human BLs [50], while transcription factors Myb and BACH2 regulate expression of many different GC B cell genes and are required for formation of the GC [51,52]. BACH2 also turns off BLIMP1 (PRDM1) expression, thereby blocking plasma cell differentiation [52]. In contrast, cyclin D2, which is expressed in EBV-transformed LCLs but not in EBV+ BLs [49], is turned down in the ΔEBNA2 + Myc lymphomas (**Fig 5A**). Interestingly, the one lymphoma infected with ΔEBNA2 + Myc that still expresses LMP1 (tumor #8 in **Fig 5A**) expressed cyclin D2 but not cyclin D3, suggesting that loss of LMP1 expression contributes to the cyclin D2 to cyclin D3 switch. Nevertheless, the ΔEBNA2 + Myc lymphoma cells do not express the master regulator of GC differentiation, BCL6 (**S3 Fig**) and thus are not true GC B cells. Together, these results reveal that high level Myc expression in naïve B cells activates expression of many cellular genes normally associated with the GC B cell differentiation state even in the absence of BCL6 expression, particularly those genes expressed in the GC “dark zone”.

### Myc-expressing lymphomas have enhanced expression of the DNMT3B and UHRF1 proteins

Although human BLs are globally hypo-methylated, specific cellular genes, including genes activated by NF-κB and STAT signaling, are hyper-methylated in human BL tumors [53]. The RNA-seq results (**S1 Table**) revealed that the *de novo* DNA methyltransferase gene DNMT3B and the UHRF1 gene (required for function of the maintenance DNA methyltransferase, DNMT1) are expressed at higher levels in the Myc-expressing tumors, and this result was confirmed at the protein level using immunoblot analysis (**Fig 5D**). Expression of DNMT1 is also generally increased in the Myc-expressing tumors (**Fig 5D**) at the protein level. UHRF1 is expressed at extremely high levels in normal GC-type B cells and is required for their proliferation [54], while DNMT3B and DNMT1 are highly expressed in human BLs in a Myc-dependent manner [55]. Furthermore, DNA methylation-mediated repression of LMP1 expression in EBV-superinfected Burkitt lymphoma cell lines *in vitro* is initiated by the DNMT3B *de novo* methyltransferase and maintained by the DNMT1/UHRF1 maintenance methylation complex [33]. These results suggest that Myc-induced expression of DNMT3B and UHRF1 expression could play a role in inhibiting LMP1 expression in the Myc-expressing lymphomas.

### Myc-expressing lymphomas have decreased expression of NF-κB2, phosphorylated STAT3, and Src kinase

**Fig 6:**
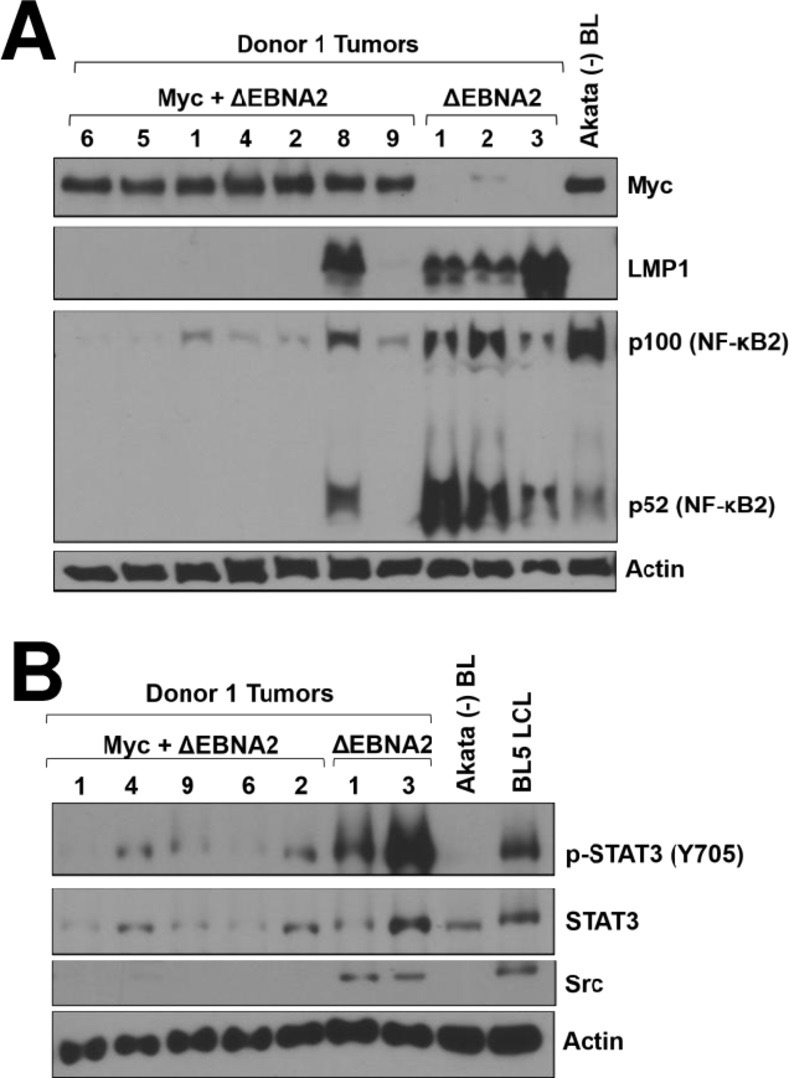
Lymphomas infected with ΔEBNA2 EBV plus Myc have decreased NF-κB2 signaling and decreased STAT3 activity compared to lymphomas with ΔEBNA2 EBV alone. **A**. Protein extracts were harvested from tumors derived with Donor 1 cells infected with ΔEBNA2 EBV alone, or co-infected with ΔEBNA2 EBV and a Myc expressing vector, and immunoblot analysis was performed to examine expression of Myc, LMP1, and p100 or its cleaved (activated) product, p52. The same extracts used in Fig 5A were used for this blot and the Myc, LMP1 and actin blots from Fig 5A are reproduced here to show levels of expression in each condition. **B.** Immunoblot analysis was performed on extracts isolated from Donor 1 tumors with or without Myc and expression of total STAT3, activated STAT3 (phosphorylated on tyrosine 705) or total Src was examined by immunoblot as indicated.

RNA-seq analysis also showed that the Myc-expressing lymphomas, like human BLs, have decreased expression of the NF-κB2 and STAT3 transcripts, as well as decreased Src kinase transcripts (**S1 Table**). Except for the one Myc-expressing tumor that also expresses high level LMP1, immunoblot analysis of tumor extracts confirmed that expression of both the full-length and cleaved (activated) forms of the NF-κB2 (p100/52) is much lower in the tumors expressing Myc (**Fig 6A**). The expression level of total Src protein, and tyrosine 705-phosphorylated STAT3 protein, is also decreased in the Myc-expressing lymphomas (**Fig 6B**) although the level of total STAT3 protein is not consistently different. Src directly phosphorylates STAT3 on tyrosine 705, allowing STAT3 to enter the nucleus and transcriptionally activate its target genes [56]. The human Akata BL cell line was also found to have much reduced Src expression and phosphorylated STAT3 in comparison to an EBV-infected LCL (**Fig 6B**). These results indicate that Myc-expressing lymphomas in this new model system have low levels of NF-κB2 and STAT3 activity, as is the case for human BLs.

### Myc-expressing lymphomas express proteins associated with the pro-B cell and pre-B cell differentiation states

**Fig 7:**
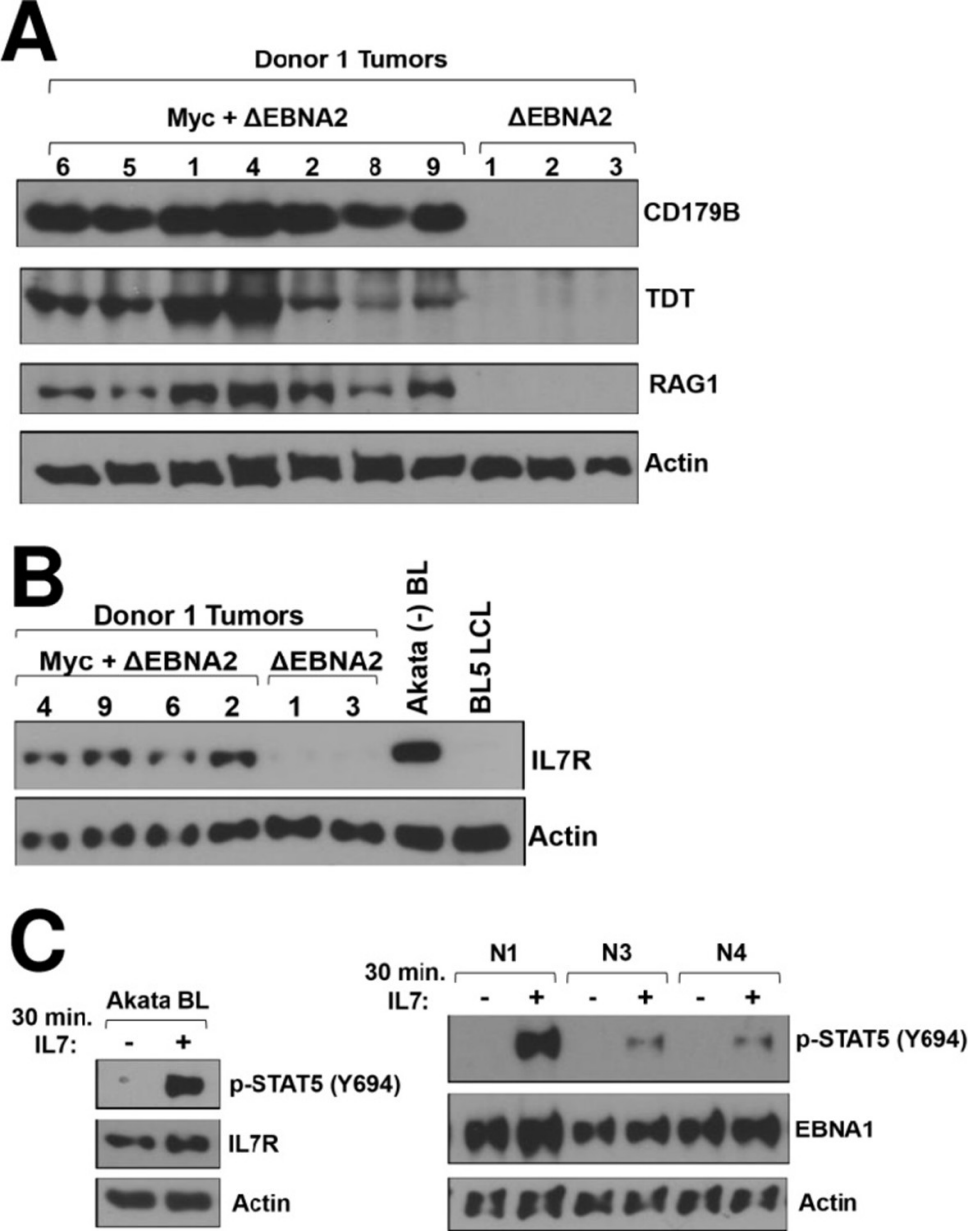
ΔEBNA2 EBV plus Myc lymphomas express cellular proteins associated with an immature B cell differentiation state. **A.** Protein extracts were harvested from tumors derived with Donor 1 cells infected with ΔEBNA2 EBV alone, or co-infected with ΔEBNA2 EBV and a Myc expressing vector, and immunoblot analysis was performed to examine expression of cellular proteins highly expressed in pro-B cells and/or pre-B cells, including CD179B (IGLL1), DNTT (TDT) and RAG1 as indicated. The extracts used are the same as those in Fig 5A with the same actin blot. **B.** Protein extracts harvested from Donor 1 derived tumors with or without Myc expression, or from an EBV-negative Akata BL cell line or an EBV strain BL5-infected LCL line, were examined by immunoblot analysis for expression of IL7R. C. Stable cell lines derived from Donor 2 ΔEBNA2 EBV plus Myc tumors (right panel), or the EBV-positive Akata Burkitt cell line (left panel) were treated with or without IL7 for 30 minutes and the level of Y694-phosphorylated STAT5 was examined by immunoblot.

**Fig 8:**
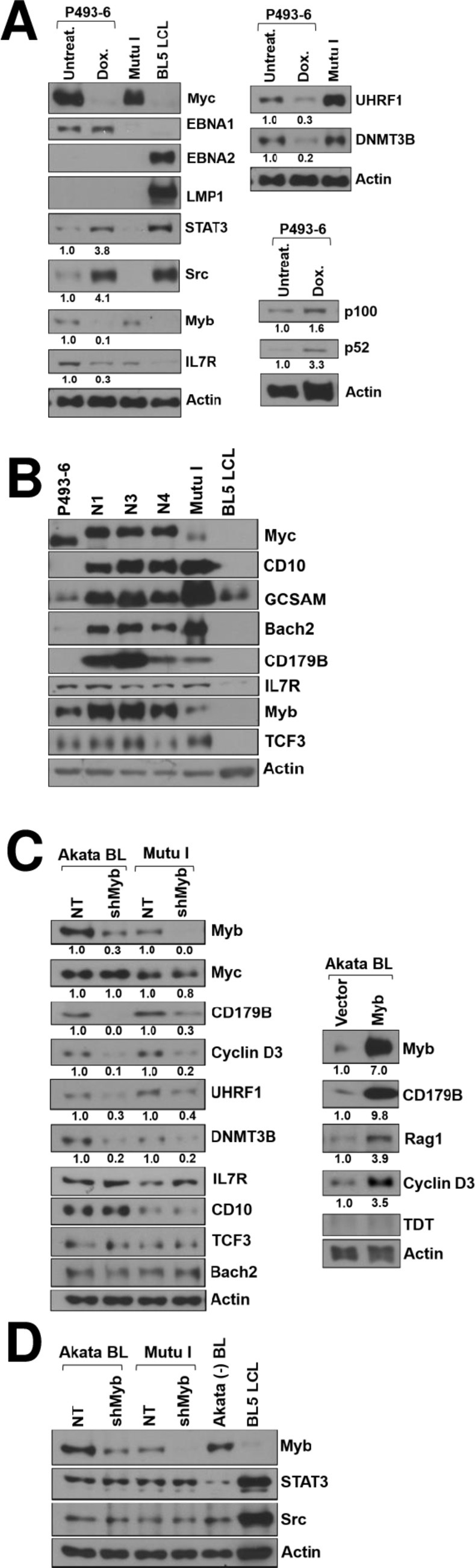
Both Myc and Myb are involved in altering expression of cellular genes in the ΔEBNA2 EBV plus Myc lymphomas. **A.** P493-6 cells were treated with or without doxycycline for 3 days, and then protein extracts were harvested to examine expression of various cellular proteins and EBV proteins as indicated. Mutu I is an EBV+ human BL cell line with type 1 latency. The blots shown on the left and top right used the same set of extracts and have the same actin blot. The numbers below each immunoblot quantify the results using Image Studio Lite software to normalize the levels of STAT3, Src, Myb, IL7R, UHRF1, DNMT3B, p100, and p52 expression to actin expression. Results are presented as the ratio of STAT3, Src, Myb, IL7R, UHRF1, DNMT3B, p100, and p52 expression in doxycycline-treated cells relative to untreated P493-6 cells, after being normalized to actin. Values of untreated cells are set as 1. The same extracts are used in S5 and S7 Figs and the actin blot is reproduced there. **B.** Immunoblot analysis was performed to compare expression of various different GC B cell proteins as indicated in stable tumor-derived cell lines (N1, N3, N4) versus the P493-6 cell line (with Myc expression turned on). The Mutu I BL line and the BL5 LCL line serve as positive and negative controls, respectively, for GC protein expression. **C.** EBV positive Akata and Mutu I BL cell lines were infected with lentiviral vectors expressing a non-targeting (“NT”) control shRNA or Myb-targeting shRNAs (left panel) or with a Myb-expressing or control lentivirus vector (right panel). Three days later, cells were treated with puromycin for 2 days prior to harvesting protein extracts for immunoblot analysis. The effect of Myb knockdown or Myb over-expression in the BL cell lines on the levels of various cellular proteins regulated by Myc expression is shown. The numbers below each immunoblot quantify the results using Image Studio Lite software to normalize the levels of Myb, Myc, CD179B, Cyclin D3, UHRF1, Rag1, and DNMT3B expression to actin expression. Results are presented as the ratio of Myb, Myc, CD179B, Cyclin D3, UHRF1, Rag1, and DNMT3B expression in Myb knockdown (or Myb over-expression) conditions relative to control conditions after being normalized to actin. Values of control conditions are set as 1. D. EBV positive Akata and Mutu I BL cell lines were infected with lentiviral vectors expressing a non-targeting (“NT”) control shRNA or Myb-targeting shRNAs and immunoblots performed to examine expression of Myb, STAT3, and Src as indicated. Akata BL line and the BL5 LCL line serve as positive and negative controls for protein expression. The same actin immunoblot will be seen in Fig 9F.

To further investigate the RNA-seq finding that genes in the “Haddad_B_Lymphocyte_Progenitor” gene set [42] are more highly expressed in the Myc-positive lymphomas (**Fig 4B**), immunoblot analysis was performed to examine protein expression of genes in this gene set that are upregulated in the RNA-seq results (**S1 Table**). In comparison to lymphomas infected with ΔEBNA2 alone, the ΔEBNA2 + Myc tumors have much higher expression of IGLL1 (CD179B, surrogate light chain), DNTT (terminal deoxynucleotidyltransferase, TDT), RAG1 and IL7R (**Fig 7A and 7B**). The expression of these proteins is normally highest in the pro-B and/or pre-B cell differentiation states, although RAG1, RAG2 and IL7R are also reported to be turned on in GC B cells undergoing light chain receptor revision [57,58]. Furthermore, we found that stable cell lines derived from the ΔEBNA2 + Myc lymphomas (grown off the feeder layer) also often express one or more pre-B cell and/or pro-B cell associated proteins (**S4 Fig**) and respond to IL7 cytokine treatment by inducing STAT5 tyrosine phosphorylation (**Fig 7C**), indicating that the IL7R is functional. IL7 has been reported to inhibit TDT expression [59] and IL7 treatment decreases TDT expression in two out of three stable tumor-derived cell lines (**S4 Fig**). Importantly, we found that some human BL cell lines also express the IL7R and respond to IL7 cytokine (**Fig 7B and 7C**), and express the surrogate light chain CD179B (**Fig 8**). Thus, GC-derived human BLs express certain genes that are normally associated with an immature B cell phenotype, but their expression of immunoglobulin light chains and germinal center associated proteins confirms their mature B cell differentiation.

### Myc and Myb collaboratively regulate cellular gene expression in Burkitt and Burkitt-like tumors

As the changes in cellular gene expression identified by RNA-seq in tumors with and without Myc could potentially be due to the effects of Myc, and/or loss of LMP1 expression, we performed experiments in the P493-6 human B cell line to identify genes regulated by Myc even in the absence of LMP1 expression. The P493-6 B cell line, which is often referred to as having a “Burkitt-like” phenotype and has been widely used to identify cellular genes turned on and off by Myc in B cells, is infected with the EBNA2-deleted P3HR1 EBV strain and contains a doxycycline-regulated Myc vector but does not express LMP1 or EBNA2 unless treated with estrogen (which turns on EBNA2 expression) [22]. As shown in **Fig 8A** (upper left panel), we confirmed that P493-6 cells express the EBV EBNA1 protein but not the LMP1 or EBNA2 proteins, allowing us to examine the effect of Myc expression on various cellular proteins without the confounding effect of altered LMP1 expression. As expected, Myc expression was dramatically decreased in this cell line after three days of doxycycline treatment. Furthermore, loss of Myc expression was accompanied not only by increased expression of the STAT3, Src and cleaved NF-κB2 proteins, but also by decreased expression of the Myb, DNMT3B and UHRF1 proteins, confirming that Myc regulates expression of each of these proteins in B cells independent of LMP1. Of note, the P493-6 cell line expresses much lower levels of some BL-associated (and GC B cell expressed) cellular proteins in comparison to the N1, N3 and N4 stable cell lines derived from the ΔEBNA2 + Myc lymphomas (**Fig 8B**), including CD10, GCSAM, BACH2, and CD179B (IGLL1). In comparison to the P493-6 line, the tumor-derived lines express similar levels of EBNA1 and EBNA3A but express much less BHRF1 (**S5 Fig**). Thus, the tumor derived cell lines derived from ΔEBNA2 + Myc lymphomas (except for the LMP1-expressing R3 line) more closely resemble human EBV+ BLs in comparison to the P493-6 cell line.

As the transcription factor Myb is expressed at very high levels in the ΔEBNA2 + Myc lymphomas (**Fig 5B**), and Myb not only serves as a master regulator of GC B cell identity and survival [51,60–62] but is also expressed at very high levels in immature B cells [63–65], we next examined whether Myc activation of Myb is required for regulation of a subset of cellular genes in human BLs. As shown in **Fig 8C and 8D**, we found that knock-down of Myb expression in the EBV+ Akata and Mutu I BL cell lines inhibits expression of CD179B (IGLL1), cyclin D3, UHRF1, and DNMT3B, although it did not affect expression of IL7R, CD10, TCF3, BACH2, STAT3 and Src. Furthermore, over-expression of Myb in the human EBV+ Akata BL cell line is sufficient to induce expression of CD179B, Rag1 and cyclin D3, although it did not turn on expression of TDT (**Fig 8C**). Together, these results indicate that Myc expression in human BL cell lines induces activation of some genes indirectly via upregulation of the Myb transcription factor.

### Myc inhibits LMP1 expression by decreasing STAT3 activity

Immunohistochemical analysis of the (rare) ΔEBNA2 + Myc lymphomas found to express both high level Myc and LMP1 on immunoblot analysis revealed that tumors are composed of geographically distinct foci containing either high level expression of Myc and Myc-induced target genes (including CD10, TDT, TCL1, and CD179B) along with very low level LMP1 expression, or high level LMP1 expression and LMP1-induced Kappa light chain expression, but low-level expression of Myc and Myc-induced target genes (**S6 Fig**). The higher-level of Kappa expression in the LMP1-high regions of tumors is consistent with the high level BLIMP1 expression in these tumors (**Fig 3A**). The finding that high level LMP1 expression and high level Myc expression do not occur in the same cells suggests that high level Myc expression inhibits LMP1 expression and/or that high level LMP1 expression is not tolerated in the tumor cells driven by high level Myc expression.

**Fig 9:**
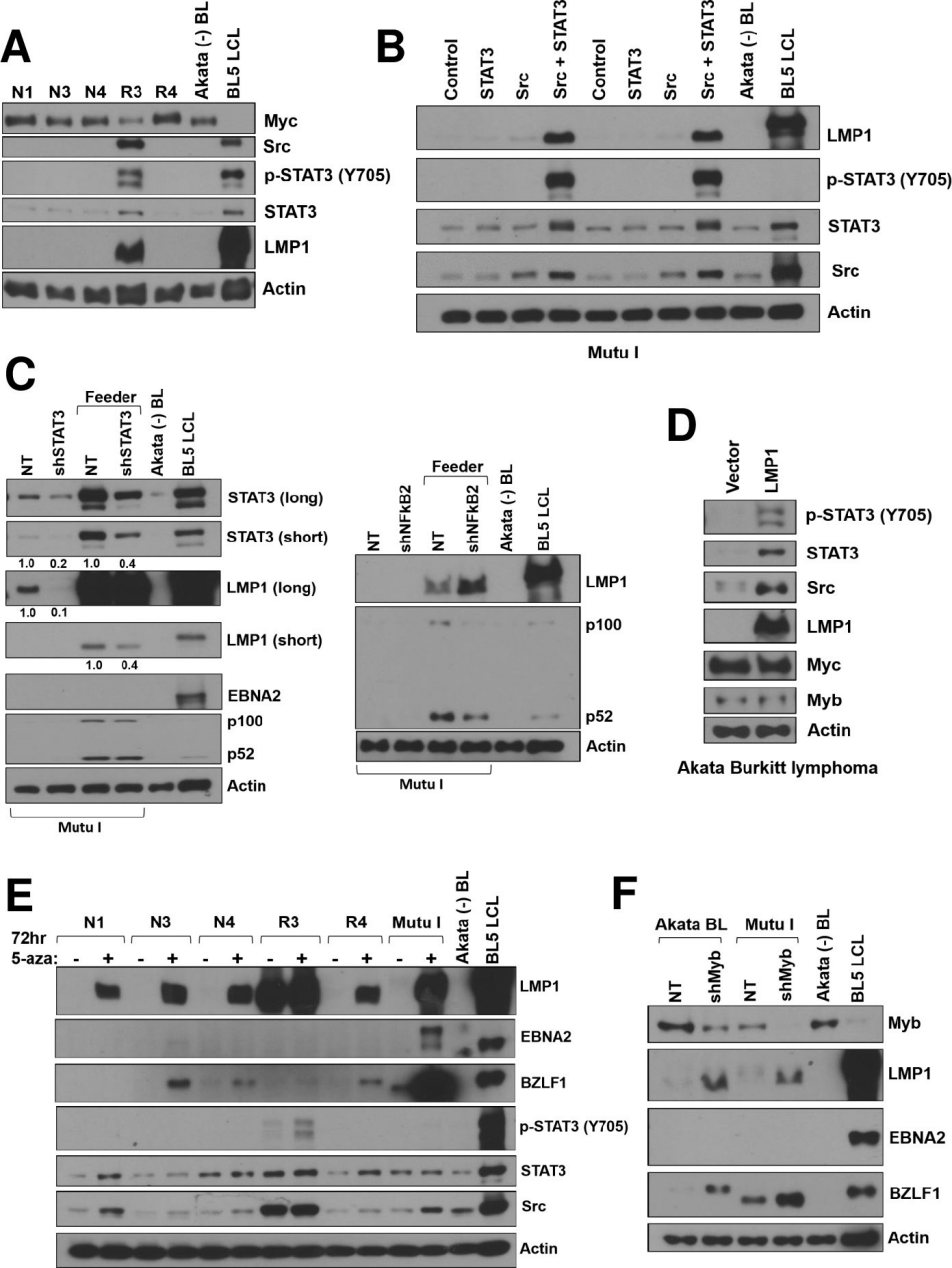
Activated STAT3 and Src regulate LMP1 expression in BL cell lines. **A**. Protein extracts were isolated from stable cell lines derived from Donor 2 ΔEBNA2 EBV plus Myc tumors (grown off the feeder layer) and immunoblot analysis was performed to examine expression of Myc, LMP1, total STAT3, phosphorylated STAT3, and total Src. The EBV negative Akata BL line and a BL5 EBV infected LCL serve as controls. **B.** Mutu I BL cells were transfected with a vector control, a STAT3 expression vector alone, a Src (constitutively active) expression vector alone, or both STAT3 and Src expression vectors. Two days later protein extracts were harvested and immunoblot analysis performed to examine expression of total STAT3, phosphorylated STAT3, Src, and LMP1 as indicated. **C.** Mutu I BL cells were infected with lentiviral vectors expressing a non-targeting control shRNA (NT) or STAT3 targeting shRNAs (left panel) or NF-κB2 (p100/52) targeted shRNAs (right panel). Three days later cells were treated with puromycin for 2 days, and then grown on or off an IL40/IL21 expressing feeder cell layer for another 2 days before harvesting protein extracts for immunoblot analysis. The levels of STAT3, LMP1, EBNA2, and p100/52 expression are shown for each condition as indicated. The numbers below each immunoblot in the left panel quantify the results using Image Studio Lite software to normalize the levels of STAT3 and LMP1 expression to actin expression. Results are presented as the ratio of STAT3 and LMP1 expression in STAT3 knockdown conditions relative to control (NT) shRNA, after being normalized to actin. Values of control conditions are set as 1. **D.** EBV+ Akata BL cells were infected with a lentivirus vector expressing LMP1 or a control lentivirus and two days later cells were treated with puromycin for 3 days. Protein extracts were harvested from puromycin resistant cells and immunoblot analysis performed to examine the levels of LMP1, total STAT3, phosphorylated STAT3, Src, and Myc as indicated. **E.** Cell lines derived from ΔEBNA2 EBV plus Myc lymphomas or Mutu I BL cells were treated with or without 5’azacitidine for 3 days and then protein extracts harvested for immunoblot to examine expression of LMP1, BZLF1, EBNA2, total STAT3, phosphorylated STAT3 or Src as indicated. F. Extracts isolated from Akata BL cells or Mutu I cells treated with non-targeting shRNA or Myb targeting shRNA (the same extracts used in Fig 8D) were examined by immunoblot analysis for expression of Myb, LMP1, EBNA2 and BZLF1 as indicated. EBV-neg Akata BL cells and a BL5 EBV-infected LCL serve as controls.

As LMP1 was reported to autoactivate its own expression in EBV-infected HeLa cells by increasing STAT3 expression [66], the ability of Myc to block LMP1 expression in BL-like lymphomas could potentially be due to Myc inhibition of STAT3 and/or Src gene expression (**Fig 6B** and **S1 Table**). Consistent with this possibility, we found that the one ΔEBNA2 + Myc tumor-derived cell line (“R3”) that constitutively expresses LMP1 when grown off the CD40L/IL21 expressing feeder cell layer also has a much higher level of total STAT3, phosphorylated STAT3, and Src in comparison to the other cell lines that do not express LMP1 (**Fig 9A**). LMP1 expression in the R3 cell line was also associated with a much lower level of Myc expression compared to the other cell lines (including the N3 cell line which was derived from the same tumor) (**Fig 9A**) as well as a lower level of Myc-induced proteins including Myb, BACH2, TCF3, and CD10 and a much higher level of p100/52 (NF-κB2) (**S7 Fig**).

To confirm that the combination of STAT3 and constitutively active Src is sufficient to induce LMP1 expression in human BL cells with type I latency, Mutu I BL cells were transfected with either a vector control plasmid, a STAT3 vector alone, a constitutively active Src vector alone, or the combination of STAT3 and constitutively active Src vectors (**Fig 9B**). Although the expression of transfected STAT3 alone was barely detectable due to the low transfection efficiency of Mutu 1 cells, the combination of STAT3 and Src together reproducibly induced LMP1 expression. In addition, we found that culturing Mutu I BL cells for two days on the CD40L/IL21 expressing feeder cell layer is sufficient to reactivate LMP1 expression (in the absence of EBNA2), and that knock-down of STAT3 expression (but not NF-κB2 expression) in Mutu I cells decreases this effect (**Fig 9C**).

Importantly, we also showed that treatment of the LMP1-expressing ΔEBNA2 + Myc tumor-derived “B1” cell line (derived from Donor 1) with the Src kinase inhibitor, dasatinib, not only strongly inhibits the level of constitutive STAT3 tyrosine 705 phosphorylation but also decreases LMP1 expression (**S8 Fig**). These results confirm that decreased Src and STAT3 expression in ΔEBNA2 + Myc tumors is an important mechanism by which Myc turns off LMP1 expression. Interestingly, in addition to decreased Src and STAT3 expression, ΔEBNA2 + Myc tumors also have altered expression levels of several other genes predicted to regulate STAT3 activity *in vivo* [67], including decreased expression of the IL21 receptor and IL21 cytokine, decreased expression of the IL10RA and IL10 cytokine, decreased expression of the IL6 cytokine and IL6R, decreased expression of the JAK1, JAK2 and JAK3 genes, and increased expression of the PTN13 and PTPRE phosphatase genes (**S3 Table**). We confirmed that loss of Myc expression in the P493-6 cell line increases IL10RA, IL21R, JAK1 and JAK2 protein expression (**S9 Fig**). Thus, Myc may also decrease STAT3 activity in human BL cells *in vivo* by decreasing their responsiveness to cytokines, including IL6, IL21 and IL10, and increasing expression of phosphatases that reverse STAT3 phosphorylation [67].

To determine if LMP1 expression at high levels is sufficient to activate STAT3 and Src expression in human BL cells, Akata Burkitt cells were infected with an LMP1 expressing retrovirus or a control vector and immunoblot analysis was performed (**Fig 9D**). LMP1 expression at high levels induces both total STAT3 and total Src expression, as well as the amount of phosphorylated STAT3, but does not alter the level of Myc expression. Thus, the relative levels of Myc versus LMP1 in B cell lymphomas likely determine whether the activated forms of STAT3 and Src are expressed. Together, these experiments confirm that STAT3 activation is sufficient to induce LMP1 expression in the absence of concomitant EBNA2 expression and strongly suggest that Myc inhibits LMP1 expression in tumors, at least partially, by decreasing expression of STAT3 and Src.

### Myc-induced DNA methylation also contributes to loss of LMP1 expression in human BLs

To examine whether Myc repression of LMP1 expression might also be mediated via enhanced DNA methylation of the LMP1 promoters, cell lines derived from the ΔEBNA2 + Myc lymphomas were treated with or without the demethylating agent, 5-azacitadine, and the levels of LMP1 expression were examined by immunoblot analysis (**Fig 9E**). LMP1 expression was increased by 5-azacitidine treatment, suggesting that enhanced DNA methylation of the proximal (ED-L1) and/or distal (TR) LMP1 promoters in the ΔEBNA2 + Myc lymphoma derived cell lines, as well as in authentic human BLs, helps to repress LMP1 expression. Furthermore, we found that knockdown of Myb expression in both the Akata BL cell line and the Mutu I BL cell line (in which DNMT3B and DNMT1/UHRF1 mediated methylation of the LMP1 promoters has been shown to inhibit LMP1 expression [33]), not only decreased UHRF1 and DNMT3B expression (**Fig 8C**) but also turned on LMP1 expression (**Fig 9F**). This result suggests that high level Myb expression in established human BLs contributes to loss of LMP1 expression by increasing DNA methylation of the LMP1 promoters via DNMT3B and UHRF1 activation. Interestingly, Myb knock-down also activated lytic BZLF1 expression in BL cells (Fig 9F), suggesting that Myb also plays a role in repressing lytic viral reactivation. Nevertheless, bisulfite sequence analysis of the proximal ED-L1 LMP1 promoter in the ΔEBNA2 + Myc lymphomas in NSG mice did not reveal CpG DNA methylation, although we could not examine the methylation state of the upstream TR LMP1 promoter due to its high GC content (**S10 Fig**). Together, these results suggest that in addition to preventing Src expression and STAT3 activation, Myc may further enforce LMP1 silencing in established human BLs by promoting DNA methylation of the LMP1 promoters.

## Discussion

A defining feature of Burkitt lymphoma (BL) is over-expression of the Myc oncoprotein, most commonly due to translocation of the Myc gene to an immunoglobulin gene locus. Nevertheless, the close association of EBV infection with pediatric BLs in sub-Saharan Africa strongly implicates EBV as a cooperating causative agent in these tumors. EBV-infected BLs support restricted forms of viral latency that do not transform B cells *in vitro* and lack expression of the major EBV oncoproteins, EBNA2 and LMP1. Here we have developed the first model system in which Myc over-expression cooperates with latent EBV infection in normal human B cells to form BL-like lymphomas in NSG mice. These lymphomas express GC type B cell proteins that are activated in (and in some cases essential for survival of) human BLs and lack both LMP1 and EBNA2 expression. In addition, we show that Myc blocks LMP1 expression both by decreasing STAT3 activity and upregulating expression of proteins that mediate LMP1 promoter DNA methylation in human BLs. Given the previously described ability of NF-κB signaling to inhibit the formation and survival of EBV-negative Myc-induced BLs [28,29], inhibition of LMP1-induced NF-κB activity may likewise be essential for the development of EBV+ human BLs.

Over-expression of Myc is not sufficient to induce Burkitt-like B cell lymphomas, as high level Myc expression not only activates growth-promoting cellular genes but also enhances expression of pro-apoptotic genes. Thus, B cell lymphomas induced by transgenic Myc over-expression in mice commonly contain inactivating mutations in cellular genes such as TP53 (p53) that induce apoptosis [68]. In addition, specific mutations in the Myc gene itself that reduce the ability of Myc to activate expression of the pro-apoptotic BIM protein are also selected for in both transgenic Myc mouse models and human BLs [11,12]. In our experiments here, we did not obtain cord blood B cell lines stably infected with Myc-expressing vectors (even using the CD40L/IL21- expressing feeder layer) unless the cells were first infected with the EBNA2-deleted EBV mutant. Similar studies performed using human primary tonsil GC B cells and the same feeder layer system likewise found that stable Myc-expressing cell lines could not be obtained when cells were infected with a Myc expressing retrovirus alone, presumably due to Myc-induced apoptosis, but could be obtained when cells were co-infected with both a BCL2- and Myc-expressing retrovirus [38]. These results suggest that infection of B cells with the EBNA2-deleted EBV mutant protects cells from Myc-induced apoptosis. The finding here that tumors over-expressing wild-type Myc (Donor 1 derived tumors) do not have Myc-activated BIM expression in comparison to the tumors infected with the ΔEBNA2 EBV alone suggests that EBV genes and/or RNAs prevent Myc-induced BIM expression, possibly through the previously described ability of the EBNA3A/3C proteins to inhibit BIM expression [15]. Furthermore, the presence of wild-type p53 in the Myc-expressing tumors suggests that viral genes/RNAs reduce selection for p53 mutations. Whether EBNA3A/EBNA3C expression can eventually be turned off in long-term tumor-derived cell lines (perhaps in conjunction with epigenetic-mediated repression of BIM expression) and whether other EBV-encoded proteins and/or RNAs are also required for survival of the tumor-derived cell lines, will be an important area for future studies.

Advantages of the model system we describe here (in which normal human cord blood B cells are infected with an ΔEBNA2 EBV mutant, and Myc over-expression is subsequently induced) in comparison to transgenic Myc and EBV gene over-expression models in mice include the use of human versus mouse B cells, the expression of Myc and EBV proteins at levels similar to those found in human BLs, and the study of viral genes/RNA in the context of the intact EBV genome (other than EBNA2 inactivation). Furthermore, the B cell lymphomas induced in transgenic Myc over-expression models are often derived from immature B cells rather than germinal center B cells. Although our studies here used naïve cord blood B cells rather than germinal center B cells to induce lymphomas, the model could be easily altered to use primary tonsil GC B cells, as recently described in a model system for human DLBCL tumors [38].

Nevertheless, our use of cord blood B cells instead of GC B cells in these studies allowed us to discover that some cellular genes highly expressed in human BLs, previously thought to be markers of the “GC” B cell differentiation state, are instead more specifically markers of Myc over-expression. For example, we found that ΔEBNA2 EBV + Myc cord blood-derived tumors express CD10 (MME), Myb, TCF3, BACH2, GCSAM, TCL1, CCND3 (cyclin D3), DNMT3B and UHRF1, even in the absence of BCL6 expression (often considered the master regulator of GC cell identity), suggesting that the high-level expression of these genes in human BLs is not necessarily due to the GC differentiation state per se but rather Myc over-expression. Furthermore, using the P493-6 cell line, in which Myc expression can be turned off by doxycycline treatment, we confirmed that expression of most of these genes is induced by Myc. In addition, using this new model system for BL, we discovered that high level Myc expression in human cord blood B cells induces expression of cellular genes normally expressed at maximal levels in immature B cells, including IGLL1 (CD179B), IL7R, RAG1 and DNTT (TDT), and that in some cases this effect is mediated by Myc-induced expression of the Myb transcription factor. Likewise, we found that Myc activation of a subset of genes normally associated with a GC B cell phenotype, including CCND3, DNMT3B and UHRF1, also requires Myb activity. Myb, a master regulator of both GC B cell and pro-B cell and pre-B cell differentiation [65,69], is required for survival and proliferation of human BL cells [51]. Based on the results obtained with the new model system, we discovered that authentic human BL cell lines, although clearly of GC B cell origin, also express both IGLL1 (a surrogate light chain normally expressed in pre-B cells) and IL7R (an essential survival receptor normally expressed in pro-B cells), and that at least some of the Burkitt cell lines retain the ability to respond to the IL7 cytokine. Whether IL7-induced signaling or surrogate light chain expression plays any role in supporting the growth of human BLs in patients is an interesting area for future study.

The model system described here is in some respects similar to the P493-6 *in vitro* cell system described by the Kempkes lab [21,22]. Importantly, both models reveal that latent EBV infection cooperates with Myc to transform normal human B cells in the absence of EBNA2, LMP1 and LMP2A expression. Differences in EBV gene expression in the two models include the lack of BHRF1 expression in the new model, and the expression of full length rather than partially deleted EBNA-LP. Thus, it appears that neither BHRF1 nor the truncated form of EBNA-LP is required for the ability of Wp-restricted EBV latency to cooperate with Myc to transform B cells. Another difference between the two models is stronger expression of GC B cell proteins (including CD10, BACH2, GCSAM and MYB) in the new model, and expression of progenitor B cell proteins (including CD179B, RAG1 and TDT) only in the new model. The stronger expression of progenitor B cell proteins in the new model may be due to the somewhat higher level of Myb. Alternatively, as P493-6 cells underwent a sustained period of type III viral latency prior to having Myc switched on and EBNA2 turned off, one or more type III EBV proteins, particularly EBNA2, may have epigenetically modified the promoters of some cellular genes expressed in GC B cells and/or progenitor B cells such that these genes are no longer activated by Myb. Other advantages of the new model are the ability to compare the phenotypes of *in vivo* tumors with and without Myc, and to examine how LMP1 is regulated by Myc in the absence of EBNA2 expression.

The new model system described here may also be useful for studying an unusual type of human malignancy described by the WHO as “Burkitt lymphoma/leukemia with IG-*MYC* rearrangement displaying a B-cell precursor immunophenotype”. These pre-B cell malignancies, which are driven by Myc translocations, morphologically resemble human Burkitt lymphomas but are derived from progenitor B cells rather than GC B cells and are not currently known to be associated with EBV infection [70]. Our results here suggest that EBV infection could potentially collaborate with Myc over-expression to induce this type of malignancy in humans, with EBV infection being lost once cellular mutations occur that can substitute for the role of EBV.

The finding here that almost all Myc-expressing BL-like tumors in this new model system lose LMP1 expression is reminiscent of previous studies describing the inhibitory effect of NF-κB signaling on Myc-induced BL tumors in mice [28,29], and suggests that EBV-infected Myc-driven BLs cannot occur unless LMP1 expression is turned off. In contrast to our results here, LMP1 was recently reported to cooperate with the transgenic λ-Myc mouse model [71] to produce B cell lymphomas [72]. NF-κB signaling driven by constitutive CD40 activation also cooperates with transgenic λ-Myc to induce lymphomas, but the tumors derived are aggressive activated DLBCL-like lymphomas rather than BLs [73]. Thus, the relative levels of Myc versus NF-κB signaling factors (and perhaps the timing of Myc over-expression in B cells) may determine whether Myc and NF-κB collaboratively induce NF-κB driven DLBCL-like lymphomas or instead NF-κB inhibits Myc-driven BL-like lymphomas in transgenic mouse models. It will be important to investigate further whether mouse lymphomas driven by LMP1/λ-Myc in transgenic mice are primarily LMP1/NF-κB-driven DLBCLs versus Myc-driven BLs. In any event, there is little evidence that LMP1 protein is expressed in latently infected primary human BL cells, although the enhanced level of LMP1 expression that occurs in lytically infected B cells may complicate analysis of LMP1 expression using RNA-seq generated data.

Finally, the studies here also reveal how Myc powerfully blocks LMP1 expression in B cells with type II latency, in which EBNA2 is not expressed. We show that Myc-mediated reduction in STAT3 and Src kinase expression plays a key role in turning off LMP1 expression in human BLs. Furthermore, we demonstrate that LMP1 expression in human BL cells with type I latency is sufficient to turn on expression of STAT3 and Src kinase and to induce high levels of Y705-phosphorylated STAT3. Thus, our results suggest a model in which LMP1 activates its own transcription in cells with type II latency by enhancing STAT3 activity, and Myc over-expression blocks this effect by reducing expression of STAT3 and Src. Consistent with our results here, prior work demonstrated that EBV-positive Burkitt lymphoma cells lack the canonically active Y705 p-STAT3 [74]. Interestingly, although high level expression of unphosphorylated STAT3 decreases induction of lytic EBV infection in Burkitt cells *in vitro* [23,74], we found here that the Myc-expressing tumors have a lower level of EBV lytic gene expression (S2C Fig) despite their decreased STAT3 levels. Our finding that Myc-expressing tumors have decreased lytic gene expression is consistent with a previous study showing that Myc decreases lytic EBV reactivation in Burkitt cells by binding to the EBV origin of lytic replication and inhibiting its looping to the BZLF1 promoter [40].

In addition to the reduced levels of STAT3 and Src expression in the Myc -expressing tumors, our RNA-seq results revealed that the Myc-expressing tumors also have reduced expression levels of the IL21, IL21R, IL10, IL10RA, IL6, IL6R and JAK 1–3 genes (S3 Table), suggesting that BL tumor cells in humans may be at least partially impaired for the ability to induce STAT3 activation in response to various cytokines, including the T cell-generated cytokines IL21 and IL10. Of note, the ability of high level Myc expression to turn off JAK1 expression in B cells is also predicted to decrease type I interferon signaling. Our finding that Myc also activates expression of the DNMT3B and UHRF1 proteins, which are known to inhibit LMP1 expression in human BL cells by increasing methylation of the LMP1 promoters [33], suggests an additional mechanism by which Myc represses LMP1 expression in fully established BLs. Importantly, because LMP1 expression in B cells potently enhances T cell recognition and killing of tumor cells through a variety of different mechanisms [75], restoring LMP1 expression in human BL tumors is predicted to be a powerful approach for enhancing the host immune response to these tumors. Our results here suggest that cytokine therapies such as IL21 (which enhances JAK kinase-mediated STAT3 activity) may be useful for reactivating LMP1 expression in human BLs, particularly when combined with epigenetic modifiers such as HDAC inhibitors and/or DNA methylation inhibitors.

## Methods

### Ethics statement

All animal work experiments were approved by the University of Wisconsin-Madison Institutional Animal Care and Use Committee (IACUC) and conducted in accordance with the NIH Guide for the care and use of laboratory animals (protocol numbers M005197 and M005214).

### Plasmids, retroviral vectors and lentiviral vectors

All plasmid DNA was prepared using the Qiagen High-speed Midi-prep kit according to the manufacturer’s instructions. Lentiviral vectors containing shRNA targeting Myb (RHS3979-201768911, RHS3979-201768912, RHS3979-201768914, RHS3979-201768915, RHS3979-201786168, RHS3979-201799542) and STAT3 (RHS3979-201751956, RHS3979-201751957, RHS3979-201751958, RHS3979-201751959, RHS3979-201751960) on a pLKO backbone were obtained from Horizon Discovery (Lafayette, CO, USA). The following plasmids were purchased from Addgene, (Watertown, MA): pMSCV-Myc-GFP was a gift from Scott Lowe (Addgene plasmid #18770; http://n2t.net/addgene:18770; RRID:Addgene 18770), pMSCVpuro-Flag-cMyc T58A was a gift from Juan Belmonte (Addgene plasmid #20076; http://n2t.net/addgene:20076; RRID:Addgene_20076), psPAX2 was a gift from Didier Trono (Addgene plasmid #12260; http://n2t.net/addgene:12260; RRID:Addgene: 12260), phCMV-GALV-MTR was a gift from Daniel Hodson (Addgene plasmid #163612; http://n2t.net/addgene:163612; RRID:Addgene_163612), TFORF2072 (Myb expression vector) was a gift from Feng Zhang (Addgene plasmid #141905; http://n2t.net/addgene:141905; RRID:Addgene_141905), Src-Y530F was a gift from Cheng-han Yu (Addgene plasmid #124659; http://n2t.net/addgene:124659; RRID:Addgene_124659), and CA-Stat3-t2A-mCherry was a gift from Jennifer Mitchell (Addgene plasmid #127540; http://n2t.net/addgene:127540; RRID:Addgene_127540). The plasmid pSG5 was purchased from Stratagene. pSG5-R and pSG5-Z (kind gifts from Diane Hayward of John Hopkins University) contain the BZLF1 (Z) and BRLF1 (R) immediate-early genes driven by the SV40 promoter as previously described [76]. The pCDH-MSCV-MCS-EF1-GFP-puro lentivirus vector is from System Biosciences (Palo Alto, CA). A LMP1-expressing lentiviral vector was constructed by inserting the B95.8 EBV strain LMP1 gene into the pCDH-MSCV-MCS-EF1a-GFP-Puro vector (CD713-LMP1). The pcDNA3 vector is from Invitrogen, (Waltham, MA). pMD-Gag-Pol was the kind gift of Richard Mulligan (Harvard University) via Robert Kalejta (UW-Madison) [77]. The CRISPR/CAS 9 vector pCEP has been described previously [78].

### Cell lines and cell culture

The EBV-positive Akata Burkitt lymphoma cell line was originally derived by the Takada lab [79]. The EBV Akata-BX1 Burkitt lymphoma cell line was a gift from Lindsay Hutt-Fletcher and was obtained by super-infecting an EBV-negative clone of Akata Burkitt lymphoma cells with recombinant Akata EBV containing a G418 resistance gene cassette and GFP gene inserted into the EBV BXLF1 gene as previously described) [80]. Type 1 EBV infected Mutu I, originally derived by the Rickinson lab at the University of Birmingham, UK [81], is a Burkitt lymphoma cell line (obtained as a gift from Jeff Sample at Pennsylvania State University). EBV-negative Akata Burkitt lymphoma cells were a kind gift from Kenzo Takada of Hokkaido University, Japan, via Bill Sugden of the University of Wisconsin. The P3HR1 Burkitt lymphoma G668 (Cl 16) cell line was a gift from George Miller at Yale University via Bill Sugden at UW-Madison [82]. All Burkitt lymphoma cell lines were maintained in RPMI media (Gibco) containing 10% fetal bovine serum (FBS), and 1% penicillin-streptomycin (pen/strep, Gibco); Akata-BX1 cells also were treated with 500 μg per ml of G418 (Life Technologies). Lymphoblastoid cell lines (LCLs) were created as previously described [83]; briefly, adult peripheral blood mononuclear cells were isolated by Ficoll gradient and EBV infected LCL lines were obtained by transforming peripheral blood B with EBV. LCLs were grown in RPMI media containing 10% FBS and 1% pen/strep. HeLa (a human cervical carcinoma cell line) was obtained from the American Type Culture Collection (ATCC, Rockville, MD) and grown in DMEM media (Gibco) with 10% FBS and 1% pen/strep. 293 FT cells (ATCC) were maintained in DMEM media (Gibco) with 1mM sodium pyruvate (Gibco), 1X non-essential amino acids (Gibco), 10% FBS, 500 μg per ml of G418, and 1% pen/strep. YK6-CD40lg-IL21 feeder cells were cultured as previously described [38]. Confluent cells were trypsinized and gamma-irradiated to 30 gys, centrifuged and frozen in 10% DMSO/FBS. For use, cells were thawed, diluted with Advanced RPMI (A-RPMI, Gibco) and centrifuged 4 min at 400g. Supernatant was removed and cells resuspended in A-RPMI, 20% FBS, 1% Pen/Strep, and 2mM glutamine. YK6-CD40lg-IL21 were plated at a rate of 10,000 cells/cm^2^ for growth of B-cells in A-RPMI.

### Generation of B-cell lines

15x10^6^ commercial cord blood mononuclear cells (Stemcell Technologies #70007.1) were thawed and plated on prepared (irradiated) YK6-CD40lg-IL21 feeder layers and infected the following day with previously prepared ΔEBNA2 virus in a minimal volume of media at an estimated MOI of 3–6 with respect to B-cells. Cells were cultured with media changes as needed, until uninfected cultures died out, with addition of fresh irradiated YK6-CD40lg-IL21 as needed. Once lines were established, they were infected with either the pMSCV-Myc-GFP or pMSCVpuro-Flag-cMyc T58A retroviruses, generated as described below. Of note, cord blood B-cell lines infected with the ΔEBNA2 virus alone are unable to grow off the CD40L/IL21 expressing feeder cell layer and are thus not fully transformed.

### Lentivirus and Retrovirus production

For cord blood B-cell infection with retroviral vectors, the retroviral vectors were co-transfected with phCMV-GALV-MTR and pCMV-GAG-Pol vectors into 293FT cells using Lipofectamine 2000 (Thermo Fisher Scientific) as per the manufacturer’s instructions. For Burkitt cell infection with retroviral vectors, the retroviral vectors were co-transfected into 293FT cells with the pMD2.G and pCMV-GAG-Pol vectors. For Burkitt cell infection with lentiviral vectors, the retroviral vectors were co-transfected into 293FT cells with the pMD2.G and pSPAX2 vectors. The media was changed to RPMI, 10% FBS, 1X Pen-Strep the following day and lentivirus/retrovirus was then harvested at 48 hours post-transfection and 0.8 μm filtered.

### Retroviral/lentiviral infection of cord blood B cell lines

Once ΔEBNA2 EBV-infected B cell lines were established, they were infected with either the pMSCV-Myc-GFP or pMSCVpuro-Flag-cMyc T58A retroviruses (pseudotyped with the GalV-MTR coat protein in the phCMV-GALV-MTR vector), with polybrene added to the retrovirus suspension at 10 μg/ml. B cells were removed from the feeder layer by shaking, and then 2ml of the released cells were pelleted at 400g for 1 minute, The supernatant was removed and 2ml retrovirus/polybrene was added. The cells/retrovirus were then centrifuged for 90 minutes at 1400g at room temperature, and then the cells and retrovirus were added to a T-25 flask containing irradiated YK6-CD40lg-IL21 cells, and additional A-RPMI to 12.5 ml added. The cells were cultured for 2–3 weeks in the absence of any drug selection to allow outgrowth of infected cells.

### Lentiviral infection of Burkitt lymphoma cell lines

Burkitt lymphoma cells were infected with lentiviruses by adding polybrene to the lentivirus containing media at 10 μg/ml and then centrifuging BL cells with the media for 90 minutes at 1400g, room temperature. After infecting cells for 3 days, puromycin (InVivoGen, San Diego, CA) was added to the media at a concentration of 1 μg/ml and cells were harvested for immunoblot analysis 2–3 days later.

### Creation of the EBV EBNA2 mutant virus

A GFP/G418R cassette was inserted into the non-essential BXLF1 gene of the AG876 type 2 EBV strain genome in the AG876 BL cell line by homologous recombination using G418 selection, and then stably infected HeLa cell clones containing this virus were derived as previously described [84]. The CRISPR/CAS9 technique was used in the AG876 virus-infected HeLa cells to create a mutation in the EBNA2 gene that puts the reading sequence out-of-frame at EBNA2 residue 25. A pCEP-based vector expressing the blasticidin resistance gene, CAS9 and an insertion site for guide RNAs was created as previously described [78,85]. The EBNA2 gene-directed guide sequence 5’-GTGGGAATTACGGAGTGA-3’was cloned into the pCEP-blasticidin vector, and 100 ng of vector was transfected into AG876 EBV infected Hela cells (in a 35 mm dish) using Lipofectamine 2000 following the manufacturer’s protocol. Twenty-four hours after transfection cells were diluted into a 100 mm dish and treated with blasticidin (Fisher/Invitrogen) at a concentration of 7.5 μg/ml. After one week of blasticidin selection, surviving cells were cultured at low confluence and individual clones were picked and grown in 24 well plates. DNA was isolated from a portion of cells in each clone using the DNAeasy Kit (Qiagen) per the manufacturer’s instructions. The EBNA2 gene sequence surrounding the guide RNA was amplified using 2x Q5 Hot-Start mastermix (New England Biolabs) and the primers 5’-GCGTGTGACGTGGTGTAAAG-3’ and 5’-AGTGTTTTCCCCGACAACGA-3’. The resulting PCR product was purified using QIAquick PCR purification Kit (Qiagen) and sequenced by GeneWiz (South Plainfield NJ). HeLa cell clones containing only the desired EBNA2 mutation and no detectable wild-type AG876 EBNA2 sequence were transfected with expression vectors for the EBV immediate-early proteins, SG5-BZLF1 and SG5-BRLF1, to reactivate lytic EBV infection in 10cm dishes. The following day, the cells were expanded to 15 cm plates and virus production allowed for 3 days. The media was then filtered through a 0.8 μm filter and the virus concentrated by ultracentrifugation in an SW28 rotor at 12,000 RPM, 4°C for 3 hours. The supernatant was decanted and the virus resuspended overnight in RPMI, then aliquoted and stored in liquid nitrogen. The DNA sequence and EBNA2 protein sequence produced by the CRISPR/CAS9 mutagenesis is shown in **Fig 1A.**

### Infection of NSG mice with EBV-positive Cord blood cell lines and tumor analysis

To generate tumors, B-cells infected with the ΔEBNA2 virus or the ΔEBNA2 virus with c-Myc retrovirus were expanded on feeder layers, and then cells were removed from the feeder layer, concentrated and 10x10^6^ cells/mouse were resuspended in 100 μL ice cold PBS and 100 μL Geltrex (Gibco), then injected into the subcutaneous space on the right flank of NSG mice. Mice were monitored weekly to twice weekly for tumor development. When tumors reached 10mm+ size, mice were euthanized with CO_2_, and if the tumors were to be cultured, the flank of the mouse sterilized with 70% ethanol, the tumor harvested sterilely, and a portion transferred to A-RPMI, with the balance of the tumor harvested, split and preserved in both 10% formalin (for histology) and frozen on dry ice (for western analysis/RNA-seq) followed by storage at -80°C. Survival data were analyzed by Kruskal-Wallis method using program M-Stat 7.0 (University of Wisconsin).

### Isolation of stable tumor-derived cell lines

To culture tumor cells, the tumor was dissociated by trituration through 10- and 5-ml pipets, and the non-dissociated fragments were allowed to settle. The supernatant containing the released cells was transferred to a fresh tube, centrifuged at 400g for 4 minutes at room temperature, the supernatant was removed, and the cells were resuspended in fresh A-RPMI media. Cells isolated from primary tumors were initially cultured for 5 days on the irradiated YK6-CD40lg-IL21 feeder layer and then reintroduced as above into NSG mice. Tumors resulting from these injections were cultured as above, and in some cases were cultured on feeder layers to amplify the cell number before adapting cells to feeder-free growth.

### Bulk RNA-seq analysis of Tumors

Bulk RNA-seq libraries were prepared as previously described [86]. Briefly, tumor cell lysates were prepared in TRIzol (Invitrogen) after powdering the tissue in liquid Nitrogen from tissue preserved on dry ice/stored at -80°C. RNA was isolated using the Direct-zol RNA MiniPrep Kit (Zymo Research) and RNA quality was assessed using an Agilent TapeStation. Sequencing libraries were prepared using the TruSeq Stranded mRNA kit (Illumina, San Diego, CA) (polyA enrichment) and sequencing on an Illumina NovaSeq 6000 with 150-bp paired-end reads was performed by the University of Wisconsin Biotechnology Center (Madison, WI). RNA-seq analysis of host transcription was conducted by BioInfoRx (Madison, WI) as previously described [86]. Briefly, fastQC was used to verify raw data quality of the Illumina reads. Reads were aligned to the GRCh38 (hg38) human genome primary assembly using Subjunc aligner from Subread [87] and assigned to genes using Ensembl annotation (v93). Raw counts were normalized using the TMM normalization method [88] using edgeR and the normalized gene counts were transformed to log2 scale using the voom method from the R Limma package [89], then used for differential expression analysis. Immunoglobulin light chain clonotypes were determined from RNA-seq results as previously described [86]. Functional interpretation of the differentially expressed genes was conducted based on GO terms, KEGG pathway and GSEA [90,91] methods. On RNA-seq analysis, one tumor infected with “ΔEBNA2 virus alone” was discovered to have a small amount of contamination with cells from the same donor that had been infected with ΔEBNA2 virus plus a retroviral vector expressing Myc and Bcl2. The RNA-seq results obtained from this tumor were removed from the analysis comparing the RNA-seq results of Myc positive versus Myc negative tumors. However, since the amount of Myc protein contamination in the tumor was extremely low (see Myc expression in Tumor 2 in Fig 3A immunoblot) and only three of the “ΔEBNA2 only” tumors were large enough to obtain sufficient protein for immunoblot analysis, this “ΔEBNA2 only” tumor was included in some immunoblots shown.

### EBV gene expression analysis

The RNA-seq results obtained from the ΔEBNA2 virus plus Myc tumor 8 were removed from the analysis since this tumor expressed LMP1 (**Fig 5A**). Raw RNA-seq reads were aligned to a combined human genome (human release 32, GRCh38.p13, obtained from Gencode) and the AG876 Type 2 genome (GenBank accession number NC_009334) using STAR 2.7.6a [89]. Transcript quantification was computed using RSEM v1.3.1 [92]. This process utilized gencode v38 comprehensive gene annotations and GenBank AG876 annotation (accession number NC_009334), incorporating parameters—estimate-rspd and—strandedness reverse. The heatmap of viral transcripts was constructed by using the TPM (transcripts per million) values estimated by RSEM. Hierarchical clustering of the data was computed and visualized using the Euclidean distance method with the ComplexHeatmap (v2.8.0) R package [93].

### p53 sequence analysis

To evaluate variants, RNA-seq read files were processed through nf-core [94] rnavar release 1.0.0 with STAR 2-7-10a [95] and SnpEff 5.1d [96]. Additional variant annotations were added from Clinvar [97] using SnpSift [98]. Variants passing quality and impact filters were considered for further evaluation. No high impact (SnpEff) or Pathogenic (ClinVar) variants were found in TP53.

### Drug treatments

5-Aza-2’-deoxycytidine was purchased from Sigma (#A3565), dissolved in DMSO, and used at 5 uM. Doxycycline was purchased from Sigma (#D5207), dissolved in water, and used at 20 ng/mL of media. IL7 was purchased from PeproTech (#200–07), dissolved in 0.1% bovine serum albumin plus water, and used at 5 ng/mL of media. The Src kinase inhibitor dasatinib was purchased from Selleckchem (#S1021), dissolved in DMSO, and used at 1 uM.

### Immunoblots

Immunoblots were performed as previously described [99]. Briefly, cell lysates or tumors were combined with SUMO lysis buffer with protease inhibitors (cOmplete, Roche) and sonicated using the Q700 sonicator from QSonica. Quantitation of protein concentration was conducted with a SUMO protein assay kit (Biorad). The lysates were separated using a polyacrylamide gel and then transferred onto a nitrocellulose membrane. The membranes were subsequently blocked with 5% milk consisting of 0.1% Tween 20 and 1X PBS for one hour. Membranes were then incubated with primary antibody overnight. The following day the antibodies were removed and the membrane was washed with wash buffer (1X PBS, 0.1% Tween 20) three times for 5 minutes. The membrane was then incubated with secondary antibody suspended in 5% milk for one hour, before washing with wash buffer three times for 10 minutes before treatment with ECL (Thermo-Fisher) and imaging. Image Studio Lite software was used to quantify levels of proteins of interest relative to loading controls tubulin or actin in certain figures.

### Antibodies

The following antibodies were used for immunoblot analyses in this study: anti-R rabbit polyclonal antibody directed against the R peptide (peptide sequence EDPDEETSSQAVKALREMAD, anti-BACH2 (Cell Signaling #80775), anti-BCL6 (Cell Signaling #14895), anti-BHRF1 (Millipore #MAB8188), anti-BIM (Cell Signaling #2933), anti-BLIMP1 (Cell Signaling #9115), anti-BMRF1 (Millipore #MAB8186), anti-BZLF1 (Santa Cruz #sc-53904), anti-CD10 (Abcam #ab227659), anti-CD19 (Abcam #ab134114), anti-CD30 (Invitrogen #MA536219), anti-CD179b (Invitrogen #702901), anti-Cyclin D2 (Cell Signaling #3741), anti-Cyclin D3 (Cell Signaling #2936), anti-DNMT1 (Abcam #ab188453), anti-DNMT3B (Santa Cruz #sc-376043), anti-EBNA1 (Santa Cruz #sc-81581), anti-EBNA2 (Abcam #ab90543), anti-EBNA3A (Exalpha #F115P), anti-GCSAM (Cell Signaling #20289), anti-GFP (Santa Cruz #sc-9996), anti-IL7R (Santa Cruz #sc-514445), anti-IL10Rα (R&D #MAB2742), anti-IL21R (R&D #MAB991), anti-IRF4 (Santa Cruz #sc-56713), anti-JAK1 (Cell Signaling #3332), anti-JAK2 (Cell Signaling #3230), anti-LMP1 (Abcam #ab78113), anti-c-Myb (Cell Signaling #12319), anti-c-Myc (Abcam #ab32072), anti-p100/52 (Cell Signaling #3017), anti-RAG1 (Cell Signaling #3968), anti-SOCS1 (Cell Signaling #55313), anti-SRC (Cell Signaling #2109), anti-phospho-SRC (Tyr416) (Cell Signaling #2101), anti-STAT3 (Cell Signaling #4904), anti-phospho-STAT3 (Y705) (Cell Signaling #9145), anti-phospho-STAT5 (Y694) (Cell Signaling #9359), anti-TCF3 (E2A) (Cell Signaling #4865), anti-TCL1 (Cell Signaling #4042), anti-TDT (Invitrogen #14-9739-82), anti-UHRF1 (Cell Signaling #12387), anti-Tubulin (Sigma #T5168), and anti-Actin (Sigma #A5441). The secondary antibodies used were horseradish peroxide (HRP)-labeled goat anti-mouse antibody (Fisher #G-21040), HRP-labeled donkey anti-goat antibody (Fisher #A16005), HRP-labeled goat anti-rabbit antibody (Fisher #G-21234), HRP-labeled donkey anti-sheep antibody (Fisher #A16047), HRP-labeled goat anti-mouse light chain specific antibody (Millipore #AP200P), and HRP-labeled goat anti-rat light-chain specific antibody (Millipore #AP202P).

### Transient transfections

DNA was transfected into Mutu I and Akata BX1 cells by nucleofection using the Amaxa Nucleofector 2b device (Lonza) and program G-016 (with Buffer V) with 1–2 μg DNA per condition.

### LMP1 promoter DNA methylation analysis

DNA was isolated from tumor samples in Trizol (Invitrogen) per manufacturer’s instructions and treated with bisulfite using the EZ DNA Methylation-Direct Kit (Zymo Research, Irvine CA). DNA was amplified using 2x Q5U (New England Biolabs) and the following primers: 5’-TTAGGGTAGTGTGTTAGGAGTAAGG-3’ and 5’-CCCTCCACTTTTTTCCAAAAATA-3’. The PCR program was as follows: 98°C for 30 sec, followed by 35 cycles of 98°C for 10 sec, 62°C for 20 sec, and 72°C for 1 min, followed by a 5 minute polishing step at 72°C. PCR products were purified using the Qiaquick kit (Qiagen) and A-tailed for 30 minutes at 72°C using 5U Taq polymerase (Promega), 200 μM dATP (New England Biolabs),1x go Taq Flexi Buffer (Promega), and 400uM MgCl2 (Promega), and then ligated overnight into a pGEM-T easy vector, transformed into DH5α cells (New England Biolabs), and plated on Ampicillin/X-gal plates. Colonies bearing the appropriate insert were identified by EcoRI digest of Qiaprep Miniprep Kit (Qiagen) DNA and submitted for sequencing by GeneWiz (Burlington, MA).

## Supporting information

S1 Fig Lymphomas infected with ΔEBNA2 EBV and Myc do not express LMP2A but express EBNA-LP. Protein extracts were harvested from tumors infected with ΔEBNA2 EBV alone, or co-infected with ΔEBNA2 EBV and a Myc vector, and immunoblot analysis was performed to examine expression of the EBV LMP2A protein **(A)** or the EBNA-LP protein **(B).** The BL5 and AG876 LCL lines are a positive control for LMP2A and EBNA-LP expression and the EBV-negative Akata BL line is a negative control for LMP2A and EBNA-LP expression. The same extracts as Fig 3A were used for **(B)** and the actin blot is reproduced here.
(PDF)

S2 Fig Molecular signatures differentially regulated in ΔEBNA2 EBV alone lymphomas versus ΔEBNA2 EBV + Myc lymphomas. RNA-seq results were used to identify molecular signature gene sets that are upregulated in the Myc-expressing tumors **(A)** or down-regulated in the Myc-expressing tumors **(B)**. **C.** Heatmap of EBV gene expression levels across samples in log2-transformed Transcripts per Million (TPM). Viral genes are grouped according to their expression kinetics into Latent, Immediate-early lytic (IE), Early lytic (Early), Leaky late lytic (Leaky), or Late lytic (Late) as indicated. **D.** Immunoblot analysis was performed on protein extracts harvested from ΔEBNA2 EBV + Myc lymphomas obtained from either Donor 1 or Donor 2 to examine the level of IgM expression.
(PDF)

S3 Fig ΔEBNA2 EBV + Myc lymphomas do not express BLC6. Protein extracts were harvested from tumors infected with ΔEBNA2 EBV alone, or co-infected with ΔEBNA2 EBV and a Myc vector, and immunoblot analysis was performed to examine expression of the BCL6 protein. The EBV-negative Akata BL cell line is a positive control for BCL6 expression.
(TIF)

S4 Fig Stable cell lines derived from Donor 2 ΔEBNA2 EBV plus Myc tumors express cellular proteins associated with an immature B cell phenotype. Stable cell lines derived from Donor 2 ΔEBNA2 EBV plus Myc tumors (grown off the CD40L/IL21-producing feeder layer) were treated with or without IL7 for 48 hours and the levels of TDT, CD179B and RAG1 were examined by immunoblot.
(TIF)

S5 Fig Comparison of EBV protein expression in stable cell lines derived from ΔEBNA2 EBV + Myc lymphomas versus P493-6 Cells. Immunoblot analysis was performed to compare EBV protein expression in extracts isolated from Myc-expressing P493-6 cells versus stable cell lines isolated from ΔEBNA2 EBV + Myc lymphomas (grown off the feeder layer) as indicated. EBV-negative Akata BL cells and an EBV-infected LCL serve as positive and negative controls for EBV proteins. The same extracts were used as in Fig 8A (bottom right panel) and the actin blot is reproduced here and in S7 Fig.
(TIF)

S6 Fig ΔEBNA2 EBV + Myc lymphomas that express LMP1 on immunoblot contain geographically distinct regions of LMP1-high versus LMP1-low tumor cells that have different phenotypes. **A)** A lymphoma infected with ΔEBNA2 EBV + Myc that was found to express both Myc and LMP1 on immunoblot was paraffin fixed and examined by **A)** H & E staining, **B)** LMP1 IHC, **C)** Myc IHC, **D)** CD10 IHC, **E)** TCL1 IHC, **F)** TDT IHC, **G)** Kappa light chain IHC, **H)** Lambda light chain IHC, and **I)** CD179B surrogate light chain IHC. General regions of the tumor with high versus low LMP1 expression are indicated for each slide.
(PDF)

S7 Fig A stable LMP1-expressing cell line derived from an ΔEBNA2 EBV + Myc lymphoma has an altered phenotype compared to tumor derived cell lines that do not express LMP1. Extracts were isolated from stable cell lines derived from ΔEBNA2 EBV + Myc lymphomas (grown off the feeder layer) and immunoblot analysis was performed to compare the levels of Myc and various GC B cell markers as indicated. Only the R3 cell line expresses LMP1 (Fig 9A). The same extracts were used as in Fig 8A (bottom right panel) and S5 Fig and the actin blot is reproduced here.
(TIF)

S8 Fig Src kinase activity is required for LMP1 expression in a cell line derived from an ΔEBNA2 EBV + Myc lymphoma. A stable cell line (“B1”) derived from a Donor 1 ΔEBNA2 EBV + Myc lymphoma (grown off the feeder layer) that expresses LMP1 was treated with or without the Src kinase inhibitor dasatinib for three days (1μM), and immunoblot analysis was performed to compare the levels of EBNA1, LMP1, p-Src, total Src, p-STAT3, total STAT3, and tubulin as indicated.
(TIF)

S9 Fig Myc inhibits expression of IL21R, IL10Rα, SOCS1, JAK1, and JAK2 in P493-6 cells. P493-6 cells were treated with or without doxycycline to turn off Myc expression, and then protein extracts were harvested to examine expression of various cellular proteins as indicated.
(TIF)

S10 Fig The LMP1 ED-L1 promoter is not methylated *in vivo* in ΔEBNA2 EBV + Myc lymphomas. DNA was isolated from ΔEBNA2 EBV + Myc lymphomas or ΔEBNA2 EBV alone lymphomas, or from the EBV + Mutu I BL cell line or a wild-type AG876 EBV strain infected LCL. Bisulfite DNA methylation analysis of the proximal (ED-L1) LMP1 promoter was performed as described in the Methods. The position of the LMP1 transcription initiation site is set as 0, and sites of various CpG motifs in the promoter are indicated by circles. Black circles indicate DNA Methylation and white circles indicate no DNA methylation. Potential STAT3 DNA binding motifs are indicated.
(TIF)

S1 Table Bulk RNA-seq data of ΔEBNA and ΔEBNA2 + Myc tumors derived from Donor 1. (XLSX)

S2 Table TP53 (p53) gene variants in ΔEBNA2 and ΔEBNA2 + Myc tumors. Variants identified in RNA-Seq data and annotated from SnpEff or ClinVar as involving TP53. Filtered variants identified by rnavar were annotated by SnpEff and SnpSift (ClinVar).
(XLSX)

S3 Table Bulk RNA-seq data of ΔEBNA and ΔEBNA2 + Myc tumors derived from Donor 1 are shown for various different cellular genes known to regulate STAT3 activation. (XLSX)
