# Supplementary figures and images for: Latent Epstein-Barr virus infection collaborates with Myc over-expression in normal human B cells to induce Burkitt-like Lymphomas in mice
Source: PLoS Pathog. 2024 Apr 15;20(4):e1012132. doi: 10.1371/journal.ppat.1012132 (PMC11045125; doi:10.1371/journal.ppat.1012132)

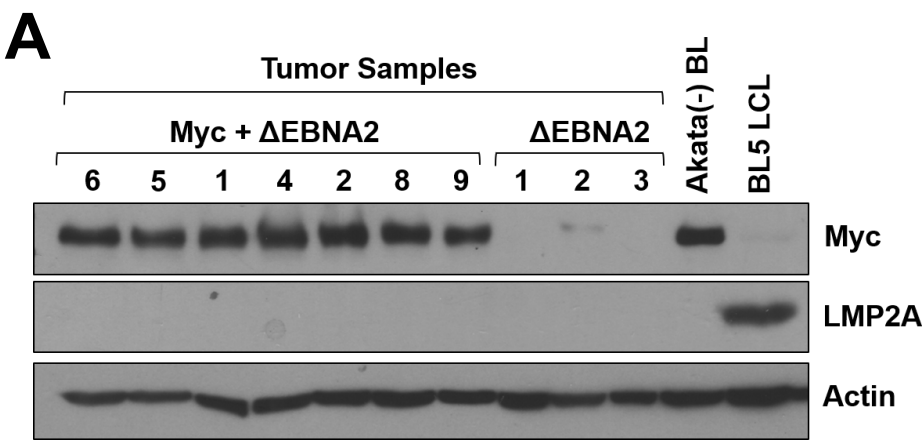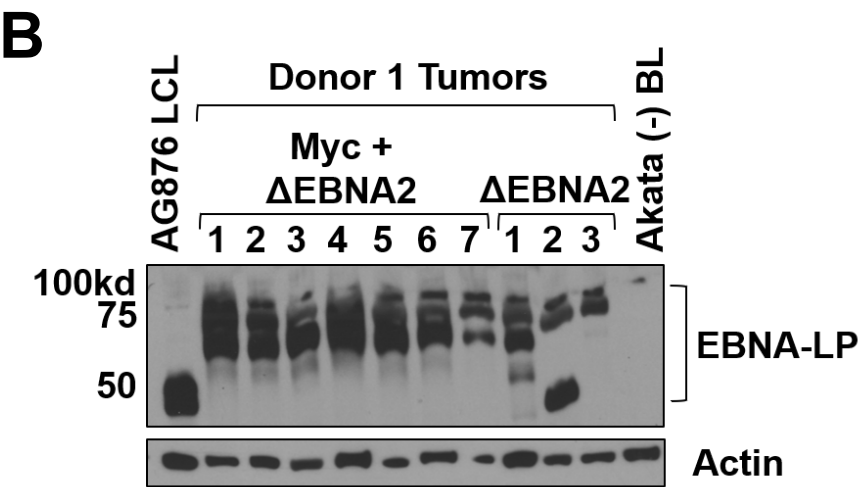

Supplement: S1 Fig — Protein extracts were harvested from tumors infected with ΔEBNA2 EBV alone, or co-infected with ΔEBNA2 EBV and a Myc vector, and immunoblot analysis was performed to examine expression of the EBV LMP2A protein (A) or the EBNA-LP protein (B). The BL5 and AG876 LCL lines are a positive control for LMP2A and EBNA-LP expression and the EBV-negative Akata BL line is a negative control for LMP2A and EBNA-LP expression. The same extracts as Fig 3A were used for (B) and the actin blot is reproduced here. (PDF) [file ppat.1012132.s001.pdf]

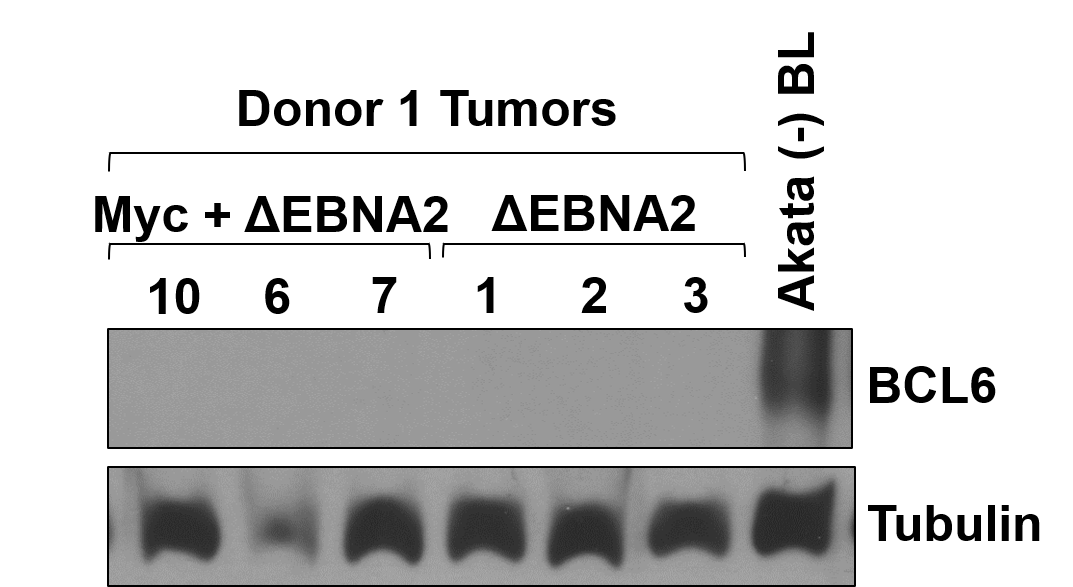

Supplement: S3 Fig — Protein extracts were harvested from tumors infected with ΔEBNA2 EBV alone, or co-infected with ΔEBNA2 EBV and a Myc vector, and immunoblot analysis was performed to examine expression of the BCL6 protein. The EBV-negative Akata BL cell line is a positive control for BCL6 expression. (TIF) [file ppat.1012132.s003.tif]

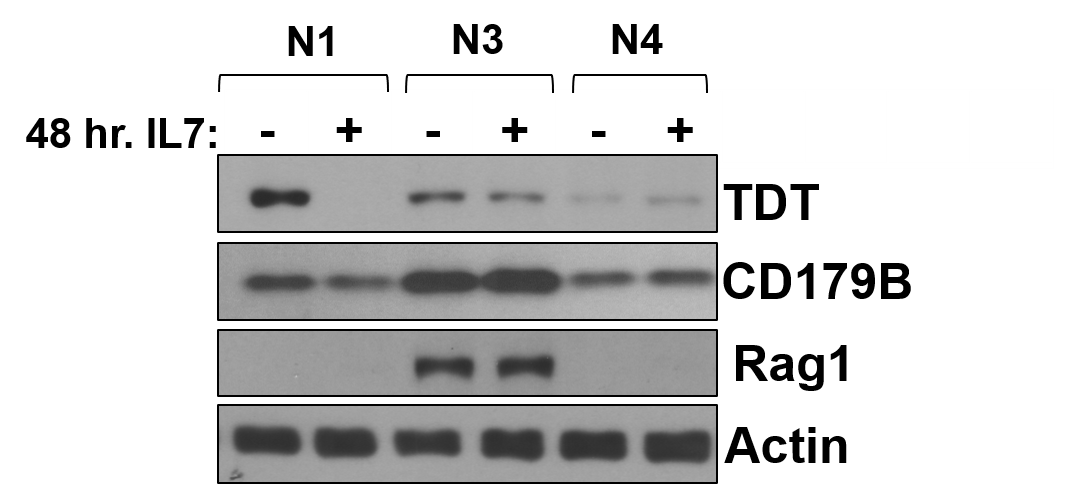

Supplement: S4 Fig — Stable cell lines derived from Donor 2 ΔEBNA2 EBV plus Myc tumors (grown off the CD40L/IL21-producing feeder layer) were treated with or without IL7 for 48 hours and the levels of TDT, CD179B and RAG1 were examined by immunoblot. (TIF) [file ppat.1012132.s004.tif]

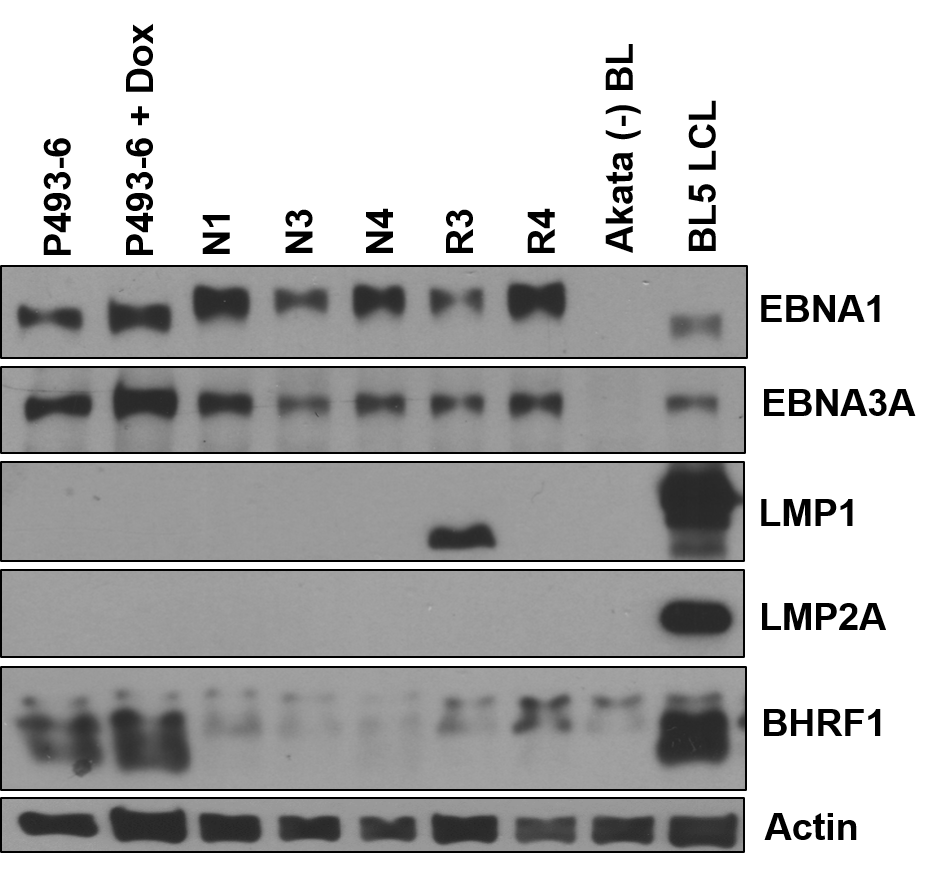

Supplement: S5 Fig — Immunoblot analysis was performed to compare EBV protein expression in extracts isolated from Myc-expressing P493-6 cells versus stable cell lines isolated from ΔEBNA2 EBV + Myc lymphomas (grown off the feeder layer) as indicated. EBV-negative Akata BL cells and an EBV-infected LCL serve as positive and negative controls for EBV proteins. The same extracts were used as in Fig 8A (bottom right panel) and the actin blot is reproduced here and in S7 Fig. (TIF) [file ppat.1012132.s005.tif]

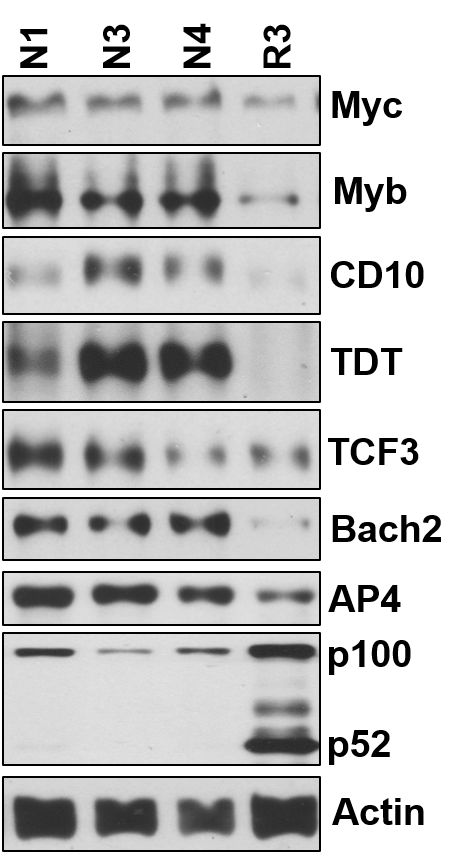

Supplement: S7 Fig — Extracts were isolated from stable cell lines derived from ΔEBNA2 EBV + Myc lymphomas (grown off the feeder layer) and immunoblot analysis was performed to compare the levels of Myc and various GC B cell markers as indicated. Only the R3 cell line expresses LMP1 (Fig 9A). The same extracts were used as in Fig 8A (bottom right panel) and S5 Fig and the actin blot is reproduced here. (TIF) [file ppat.1012132.s007.tif]

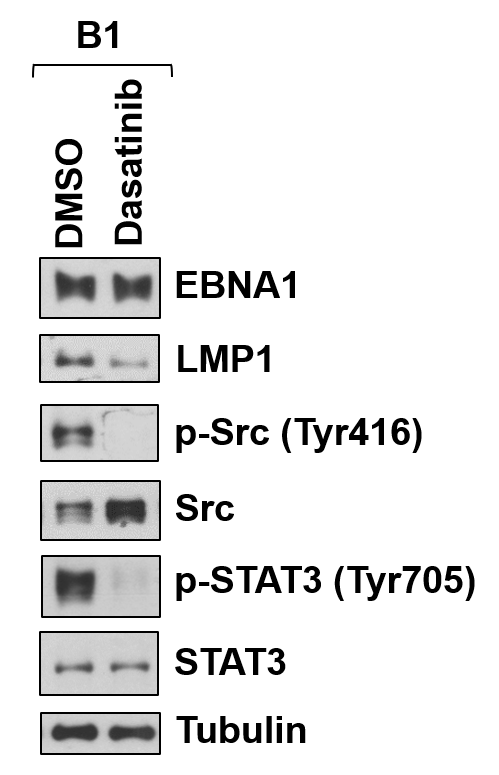

Supplement: S8 Fig — A stable cell line (“B1”) derived from a Donor 1 ΔEBNA2 EBV + Myc lymphoma (grown off the feeder layer) that expresses LMP1 was treated with or without the Src kinase inhibitor dasatinib for three days (1μM), and immunoblot analysis was performed to compare the levels of EBNA1, LMP1, p-Src, total Src, p-STAT3, total STAT3, and tubulin as indicated. (TIF) [file ppat.1012132.s008.tif]

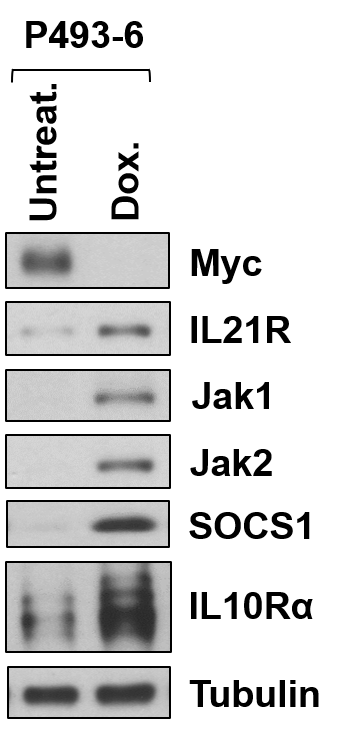

Supplement: S9 Fig — P493-6 cells were treated with or without doxycycline to turn off Myc expression, and then protein extracts were harvested to examine expression of various cellular proteins as indicated. (TIF) [file ppat.1012132.s009.tif]

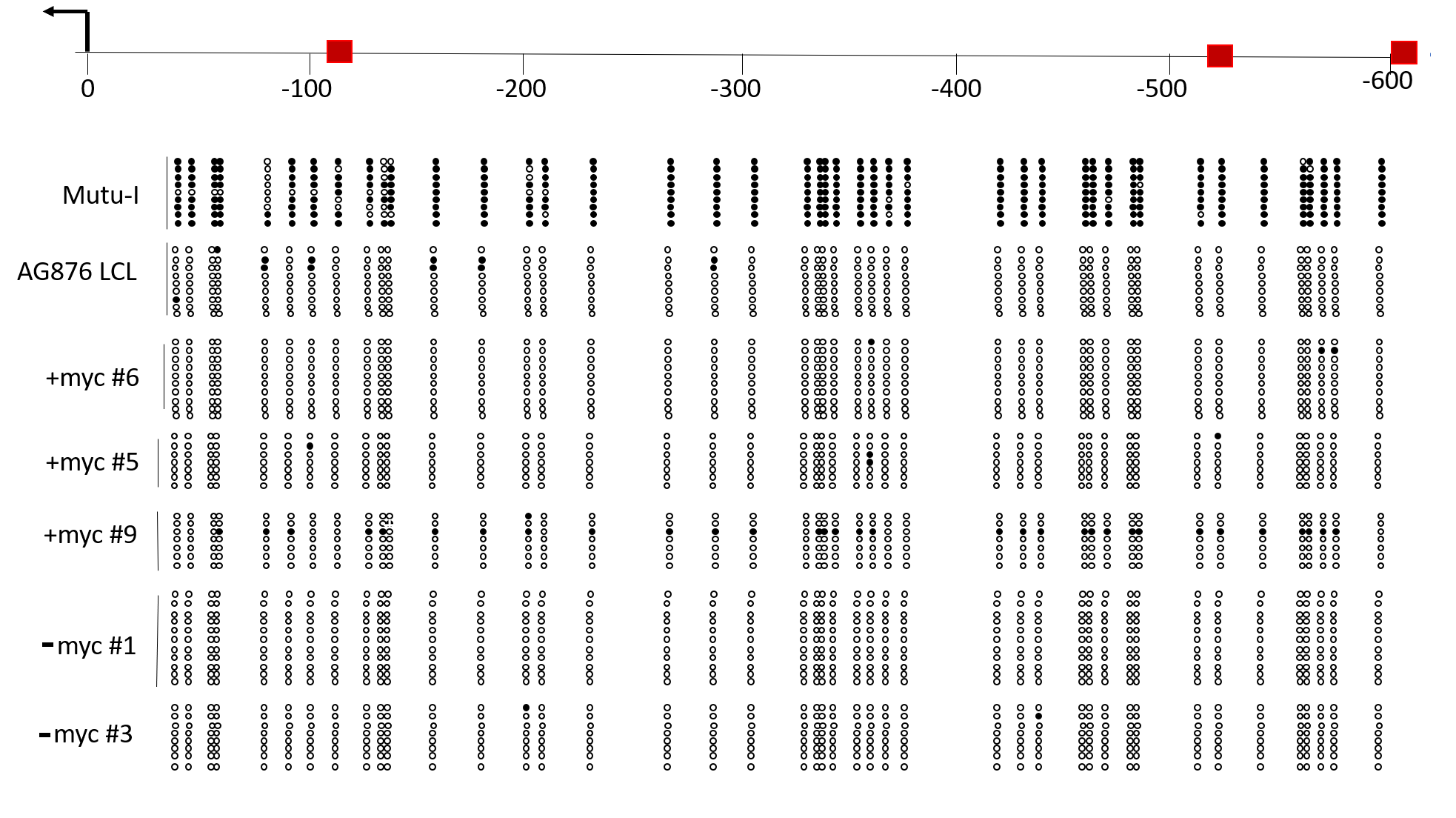

Supplement: S10 Fig — DNA was isolated from ΔEBNA2 EBV + Myc lymphomas or ΔEBNA2 EBV alone lymphomas, or from the EBV + Mutu I BL cell line or a wild-type AG876 EBV strain infected LCL. Bisulfite DNA methylation analysis of the proximal (ED-L1) LMP1 promoter was performed as described in the Methods. The position of the LMP1 transcription initiation site is set as 0, and sites of various CpG motifs in the promoter are indicated by circles. Black circles indicate DNA Methylation and white circles indicate no DNA methylation. Potential STAT3 DNA binding motifs are indicated. (TIF) [file ppat.1012132.s010.tif]
