# Supplementary material for: Latent Epstein-Barr virus infection collaborates with Myc over-expression in normal human B cells to induce Burkitt-like Lymphomas in mice
Source: PLoS Pathog. 2024 Apr 15;20(4):e1012132. doi: 10.1371/journal.ppat.1012132 (PMC11045125; doi:10.1371/journal.ppat.1012132)

A Molecular signatures upregulated in Myc-expressing tumors

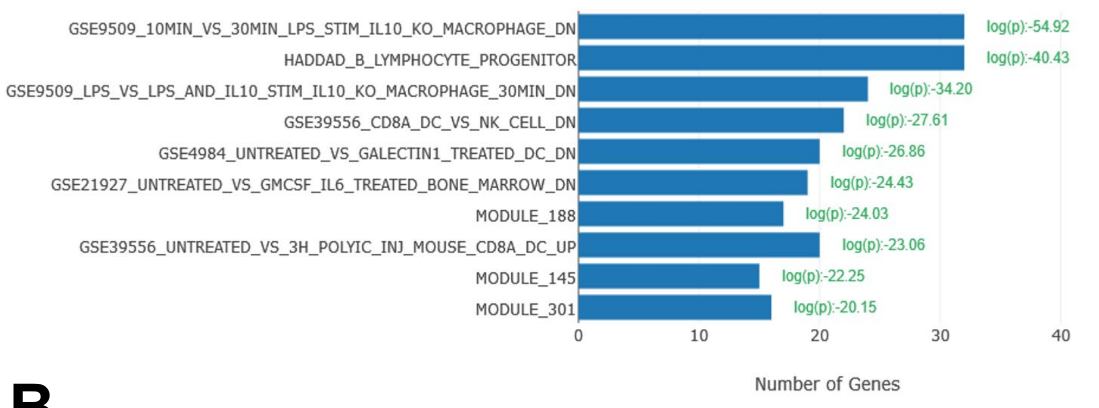

B Molecular signatures downregulated in Myc-expressing tumors

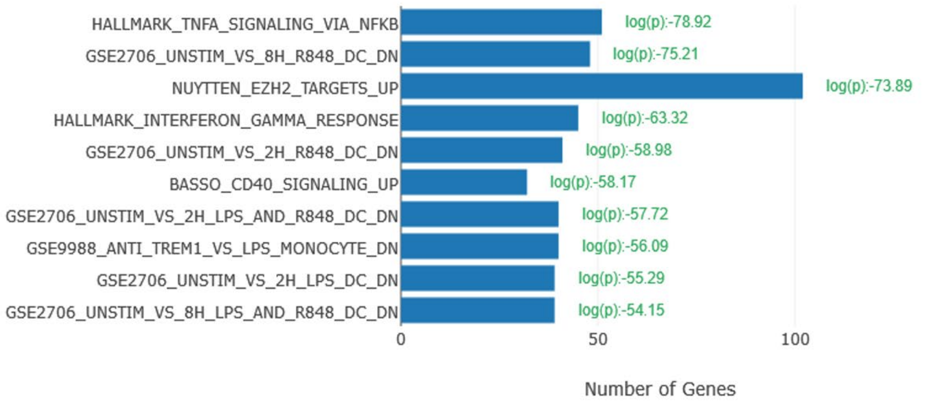

C

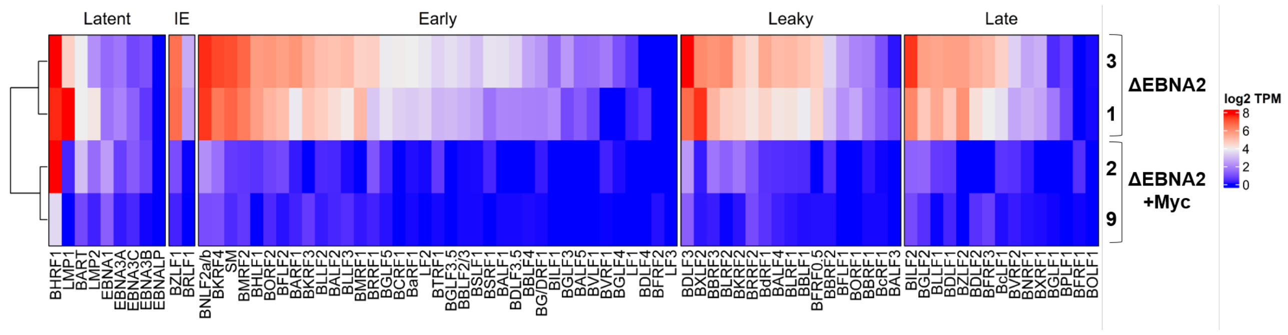

D

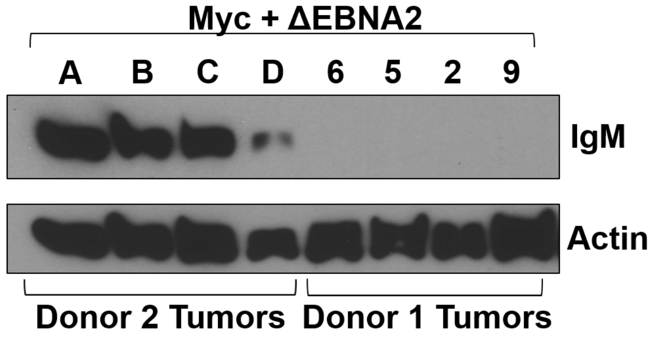

Supplement: S2 Fig — RNA-seq results were used to identify molecular signature gene sets that are upregulated in the Myc-expressing tumors (A) or down-regulated in the Myc-expressing tumors (B). C. Heatmap of EBV gene expression levels across samples in log2-transformed Transcripts per Million (TPM). Viral genes are grouped according to their expression kinetics into Latent, Immediate-early lytic (IE), Early lytic (Early), Leaky late lytic (Leaky), or Late lytic (Late) as indicated. D. Immunoblot analysis was performed on protein extracts harvested from ΔEBNA2 EBV + Myc lymphomas obtained from either Donor 1 or Donor 2 to examine the level of IgM expression. (PDF) [file ppat.1012132.s002.pdf]
