# Supplementary material for: Latent Epstein-Barr virus infection collaborates with Myc over-expression in normal human B cells to induce Burkitt-like Lymphomas in mice
Source: PLoS Pathog. 2024 Apr 15;20(4):e1012132. doi: 10.1371/journal.ppat.1012132 (PMC11045125; doi:10.1371/journal.ppat.1012132)
Supplement: S6 Fig — A) A lymphoma infected with ΔEBNA2 EBV + Myc that was found to express both Myc and LMP1 on immunoblot was paraffin fixed and examined by A) H & E staining, B) LMP1 IHC, C) Myc IHC, D) CD10 IHC, E) TCL1 IHC, F) TDT IHC, G) Kappa light chain IHC, H) Lambda light chain IHC, and I) CD179B surrogate light chain IHC. General regions of the tumor with high versus low LMP1 expression are indicated for each slide. (PDF) [file ppat.1012132.s006.pdf]

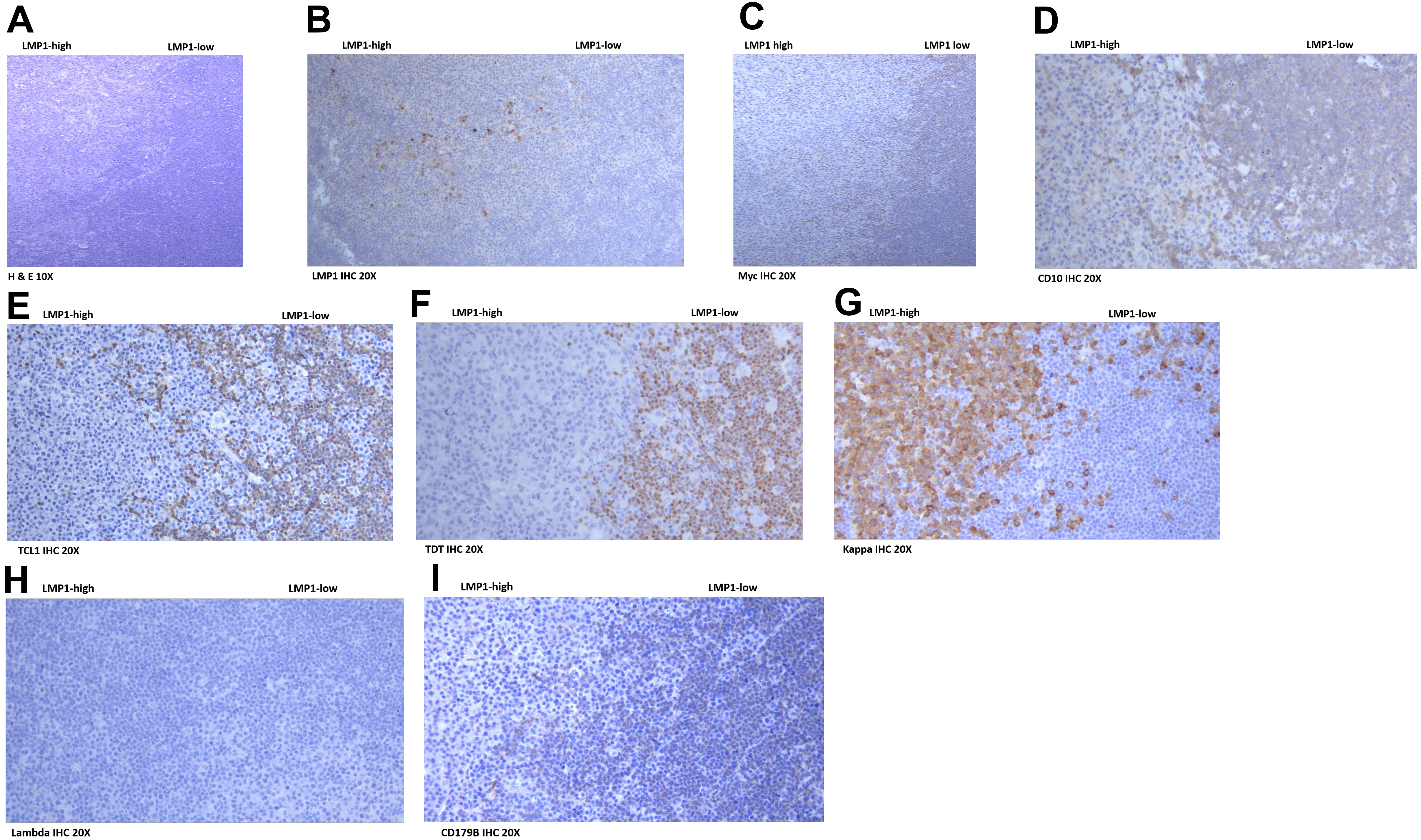
A
LMP1-high
LMP1-low
H & E 10X
B
LMP1-high
LMP1-low
LMP1 IHC 20X
C
LMP1 high
LMP1 low
Myc IHC 20X
D
LMP1-high
LMP1-low
CD10 IHC 20X
E
LMP1-high
LMP1-low
TCL1 IHC 20X
F
LMP1-high
LMP1-low
TDT IHC 20X
G
LMP1-high
LMP1-low
Kappa IHC 20X
H
LMP1-high
LMP1-low
Lambda IHC 20X
I
LMP1-high
LMP1-low
CD179B IHC 20X
